# Pyrene-Appended Boronic Acids on Graphene Foam Electrodes Provide Quantum Capacitance-Based Molecular Sensors for Lactate

**DOI:** 10.1021/acssensors.4c00027

**Published:** 2024-03-06

**Authors:** Simon M. Wikeley, Jakub Przybylowski, Jordan E. Gardiner, Tony D. James, Philip J. Fletcher, Mark A. Isaacs, Pablo Lozano-Sanchez, Marco Caffio, Frank Marken

**Affiliations:** † Department of Chemistry, University of Bath, Claverton Down, Bath BA2 7AY, U.K.; ‡ School of Chemistry and Chemical Engineering, Henan Normal University, Xinxiang 453007, China; § Imaging Facility, University of Bath, Bath BA2 7AY, U.K.; ∥ HarwellXPS, Research Complex at Harwell, STFC Rutherford Appleton Laboratory, Harwell Campus, Didcot OX11 0FA, U.K.; ⊥ Department of Chemistry, University College London, London WC1H 0AJ, U.K.; # Integrated Graphene Ltd., Euro House, Wellgreen Place, Stirling FK8 2DJ, U.K.

**Keywords:** graphene foam, quantum capacitance, lactate sensor, wearable sensor, molecular capacitance, quantum capacitance

## Abstract

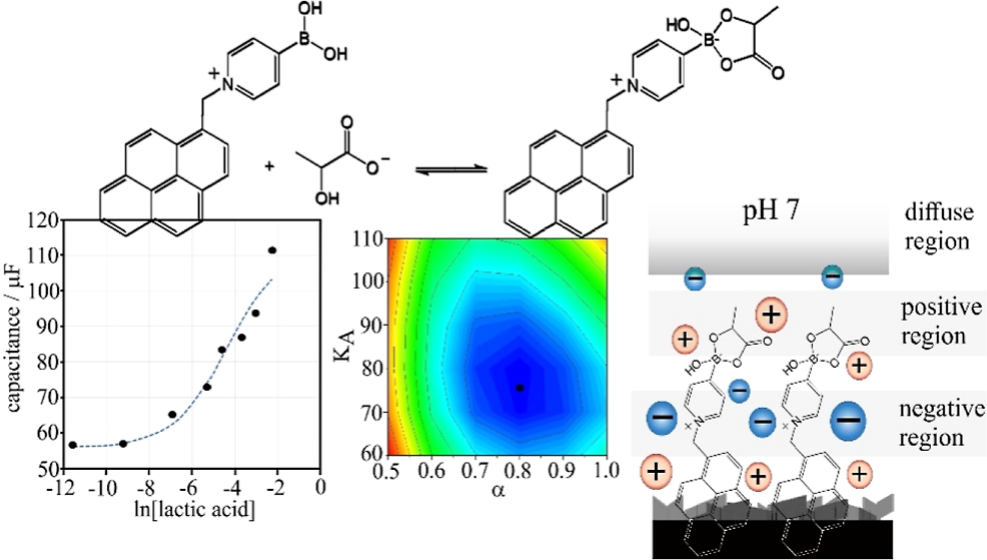

Molecular recognition and sensing can be coupled to interfacial capacitance changes on graphene foam surfaces linked to double layer effects and coupled to enhanced quantum capacitance. 3D graphene foam film electrodes (Gii-Sens; thickness approximately 40 μm; roughness factor approximately 100) immersed in aqueous buffer media exhibit an order of magnitude jump in electrochemical capacitance upon adsorption of a charged molecular receptor based on pyrene-appended boronic acids (here, 4-borono-1-(pyren-2-ylmethyl)pyridin-1-ium bromide, or abbreviated T1). This pyrene-appended pyridinium boronic acid receptor is employed here as a molecular receptor for lactate. In the presence of lactate and at pH 4.0 (after pH optimization), the electrochemical capacitance (determined by impedance spectroscopy) doubles again. Lactic acid binding is expressed with a Hillian binding constant (*K*_lactate_ = 75 mol^–1^ dm^3^ and α = 0.8 in aqueous buffer, *K*_lactate_ = 460 mol^–1^ dm^3^ and α = 0.8 in artificial sweat, and *K*_lactate_ = 340 mol^–1^ dm^3^ and α = 0.65 in human serum). The result is a selective molecular probe response for lactic acid with LoD = 1.3, 1.4, and 1.8 mM in aqueous buffer media (pH 4.0), in artificial sweat (adjusted to pH 4.7), and in human serum (pH adjusted to 4.0), respectively. The role of the pyrene-appended boronic acid is discussed based on the double layer structure and quantum capacitance changes. In the future, this new type of molecular capacitance sensor could provide selective enzyme-free analysis without analyte consumption for a wider range of analytes and complex environments.

Boronic acids provide a potent class of molecular chemical receptors with selectivity for a range of analytes including glucose^1^ and α-hydroxy-carboxylic acids.^2^ Although commonly employed in fluorescence assays,^3^ boronic acids have also been attached or assembled at electrochemical sensor surfaces,^4^ but they have never been employed directly to sense via electrochemical (quantum) capacitance responses. A related “varactor”-based quantum capacitor device with an adsorbed boronic acid on graphene was shown to respond to glucose binding.^5^ A varactor (or varicap) is a device with potential dependent capacitance. The capacitance in the varactor response was linked to double layer structure changes upon glucose binding, affecting the electronic states within the graphene. Boronic acids embedded in functional microgels^6^ have been employed for detection of lactate in sweat. Here, a special boronic acid with a positive pyridinium charge bound closely to the graphene electrode surface is introduced.

The pyrene-appended boronic acid 4-borono-1-(pyren-2-ylmethyl)pyridin-1-ium bromide or abbreviated T1 (see molecular structure in Figure 1) was developed by Tomoki Nishimura and co-workers^7^ and has been assembled, for example, onto carbon nanoparticle-modified electrodes.^8^ A precursor molecule with protected boronic acid is synthesized, attached to a graphene surface, and then employed for the sensor (see Figure 1) by neopentylglycol release in contact with aqueous media. In recent work, it was shown that T1 can be adsorbed onto graphene foam film electrodes for the voltammetric detection of glucose^9^ or for the detection of lactic acid.^10^ In these studies, a redox-active polymer was employed to modulate Faradaic current responses related to analyte binding in a polymer indicator displacement assay (PIDA). Here, it is reported that the interfacial capacitance response of 3D-graphene foam can be employed directly for sensing (without any Faradaic current consuming analyte), even in complex matrices and without the need for a redox polymer indicator.

**Figure 1:**
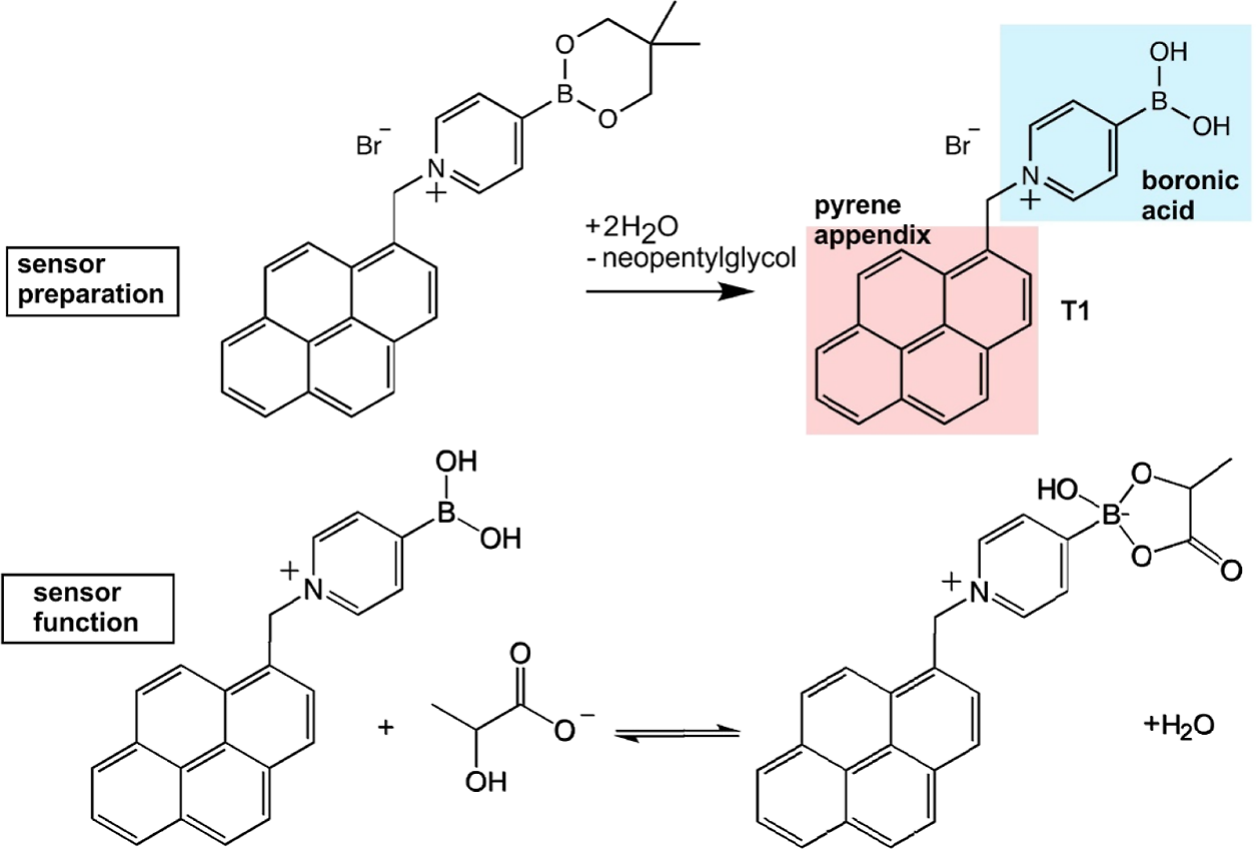
Illustration of the sensor preparation step (i) and sensor function (ii) for T1 as a pyrene-appended boronic acid.

Graphene surfaces in sensors are highly effective by combining electrical conductivity with a high surface area. Graphene foam electrodes are readily modified by attaching pyrene-appended bioreceptors.^11^ Lactate sensing on graphene has been reported for wearable devices based on lactate oxidase enzyme processes.^12^ More generally, lactate sensing has been reported based on dielectric responses^13^ and voltammetric responses, specifically based on biorecognition.^14,15^ Significant research interest in lactic acid sensing has been generated due to clinical implications (ischemia, hyperlactatemia, indicator of septic shock, and heart attack–detected early to prevent more serious condition). Other applications could include food quality control and wearable lactate monitors for medical and sporting uses. Voltammetric or amperometric sensors are lactate flux dependent and consume the analyte, but (in contrast) capacitance-based sensors only bind the lactate analyte without consuming it. Capacitance can therefore be used to monitor concentrations. Electrochemical impedance spectroscopy (EIS)^16^ offers an experimental tool to read out capacitance information. Impedance-based wireless capacitance sensors could provide competitive advantages due to no requirement for an internal power supply when reading capacitance information.

EIS has been employed successfully in many aspects of sensor development.^17^ It is employed here to monitor and/or disseminate resultant changes to interfacial capacitance at a graphene foam electrode surface upon (i) attachment of the pyrene–appended boronic acid receptor and (ii) subsequent binding of lactic acid onto the receptor. Binding of a positively charged boronic acid (T1; obtained from a precursor with molecular weight 486.21 g mol^–1^; Figure 1) increases the electrical double layer capacitance by approximately 1 order of magnitude. A further lactate concentration-dependent increase in capacitance is subsequently utilized for sensing.

It is shown here that a surface boronic acid-modified 3D-graphene foam electrode can be employed as an impedimetric lactate sensor based on capacitance, avoiding the need for less practical solution phase or immobilized redox probes for Faradaic current detection. In the future, capacitance as a sensor output parameter can be probed with an AC (alternating current) signal and without the need for an on-board power supply in microdevices (e.g., in wireless sensing^18^).

## Experimental Section

### Chemical Reagents

Chloroform (>99%), ethanol (ACS reagent), NaH_2_PO_4_, Na_2_HPO_4_, NaCl, NaOH, pyrene, NH_4_Cl, and lactic acid were obtained from Sigma-Aldrich in analytical grade purity and used without further purification. Demineralized and filtered water (ultrapure, 18.2 MΩ cm at 18 °C) was taken from a Thermo-Fisher water purification system. T1 was synthesized as reported previously.^7^ The boronic acid *N*-methyl-1-(pyren-1-yl)-*N*-(4-(4,4,5,5-tetramethyl-1,3,2-dioxaborolan-2-yl)benzyl)methanamine (abbreviated V1) was synthesized as detailed in the Supporting Information.

### Instrumentation

A computer-controlled Ivium Compactstat instrument (Ivium, The Netherlands) was employed for electrochemical measurements. Graphene foam electrodes (Gii-Sens) were obtained from Integrated Graphene Ltd (www.integratedgraphene.com). Electrochemical measurements were performed with a single droplet (volume 100 μL) placed onto the graphene foam electrode (covering the working, counter, and reference electrodes). The working electrode was graphene foam (4 mm diameter disc, approximately 40 μm thick, true surface area approximately 1.2 × 10^–3^ m^2^) with a counter electrode (1 mm ring, graphene foam), and a printed Ag/AgCl pseudo reference electrode. Scanning electron micrographs were obtained with a JEOL JSM-7900F field emission scanning electron microscope (FE-SEM) with an attached Oxford Instruments Ultim Extreme 100 mm^2^ low kV energy dispersive X-ray (EDX) analyzer. TEM images were obtained on a JEOL JEM-2100Plus instrument.

X-ray photoelectron spectroscopy (XPS) data were acquired using a Kratos Axis SUPRA using monochromated Al kα (1486.69 eV) X-rays at 15 mA emission and 12 kV HT (180 W) and a spot size/analysis area of 700 × 300 μm. The instrument was calibrated to gold metal Au 4f (83.95 eV) and dispersion adjusted to give a BE of 932.6 eV for the Cu 2p3/2 line of metallic copper. Ag 3d5/2 line fwhm at 10 eV pass energy was 0.544 eV. Source resolution for monochromatic Al Kα X-rays was ∼0.3 eV. The instrumental resolution was determined to be 0.29 at 10 eV pass energy using the Fermi edge of the valence band for metallic silver. Resolution with charge compensation system on <1.33 eV fwhm on PTFE. High resolution spectra were obtained using a pass energy of 20 eV, step size of 0.1 eV, and sweep time of 60 s, resulting in a line width of 0.696 eV for Au 4f7/2. Survey spectra were obtained using a pass energy of 160 eV. Charge neutralization was achieved using an electron flood gun with filament current = 0.4 A, charge balance = 2 V, and filament bias = 0.2 V. Successful neutralization was adjudged by analyzing the C 1s region wherein a sharp peak with no lower BE structure was obtained. Spectra have been charge-corrected to the main line of the carbon 1s spectrum set to 284.8 eV. All data were recorded at a base pressure of below 9 × 10^–9^ Torr and a room temperature of 294 K. Data were analyzed using CasaXPS v2.3.19PR1.0. Peaks were fitted with a Shirley background prior to component analysis.

### Procedures

Boronic acid-modified 3D-graphene foam electrode sensors were prepared via drop–casting of a boronic acid (T1, precursor molecular weight 486.21 g mol^–1^; equiv 2) solution (1 mg/mL, 5 μL) onto the working electrode disc, yielding a 5 μg (or 10 nmol) surface coverage. In previous work, it was shown that a lower amount of T1 decreased the sensor response and a higher amount of T1 caused blocking and loss of performance.^9,10^ The modified sensor was then rinsed using deionized water before drying and stored before use. For lactic acid sensing using EIS, a droplet (approximately 100 μL) of lactic acid in phosphate buffer at the required pH was added to a freshly prepared boronic acid-modified electrode, ensuring all electrodes were covered. The impedimetric response at 0.0 V vs Ag/AgCl was then monitored at a frequency range between 500 and 0.1 Hz. Analysis and fitting of the resulting spectra were carried out on Ivium Software using an RC-series equivalent circuit.

Serum samples were prepared by spiking an aliquot of human serum [normal, Sigma-Aldrich S1–100 ML, lot number 3764702, contains glucose (4.89 mM), BUN (0.44 mM), creatinine (0.067 mM), sodium (136 mM), potassium (4.4 mM), chloride (98 mM), calcium (0.38 mM), phosphorus (0.21 mM), uric acid (0.17 mM), albumin (0.21 M), globulin (0.12 M), bilirubin (0.02 mM), alkaline phosphatase (1 μM), LDH (4.3 μM), AST/SGOT (0.56 μM), ALT/SGPT (0.22 μM), GGTP (1.33 μM), ionized calcium (0.18 mM), iron (3.6 μM), triglycerides (3.1 mM), total protein content of 0.32 M, pH = 7.2, and no preservatives] with the prescribed amount of lactic acid, and the pH was adjusted with aqueous 1.0 M HCl. The serum pH was measured with a combination pH electrode HI-11310 (Hanna Instruments Ltd.). A droplet (approximately 100 μL) was added to the electrode, and impedimetric measurements were carried out via the same experimental protocol as in aqueous phosphate buffer. For serum samples, sensor reuse was not possible due to electrode surface contamination, meaning fabrication of a fresh sensor was necessary for each data point. For buffer and artificial sweat samples, after use, the sensor signal also did not fully recover to the baseline capacitance probably due to slow lactate desorption. Therefore, fresh sensor electrodes were employed for each data point in all cases. For artificial sweat samples, a literature composition^19^ of 327 mM NH_4_Cl, 34 mM NaCl, 83 mM urea, and 42 mM acetic acid in deionized water (adjusted to pH 4.7; sweat is naturally at pH 4.5 to 4.7) with variable concentrations of lactic acid was used as a model system to explore sensing performance in human sweat.

## Results and Discussion

### Capacitance of Graphene Foam Electrodes: Effects of T1 Adsorption

**Figure 2:**
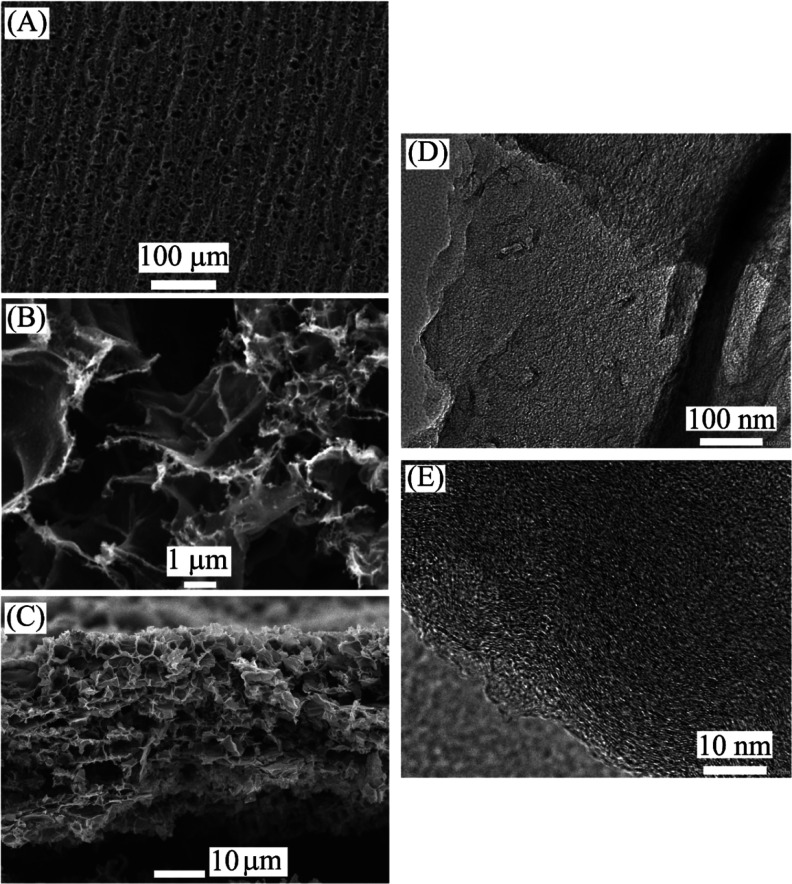
SEM images for graphene foam electrodes (A) at lower magnification and (B) at higher magnification. (C) Cross-sectional image of the graphene foam layer. TEM images for graphene foam at lower (D) and higher (E) magnification.

SEM images of 3D-graphene foam electrodes are shown in Figure 2. The porous surface and foam structure are visible with pores in the 1 to 10 μm size range. The cross-sectional image (Figure 2C) reveals an approximately 40 μm thick layer. TEM images (Figure 2D,E) reveal highly disordered multilayer graphene with approximately 2 to 10 graphene sheet wall thickness. The adsorption of T1 into the graphene foam did not affect the SEM image in terms of morphology. However, the adsorption of T1 can be verified with XPS data.

**Figure 3:**
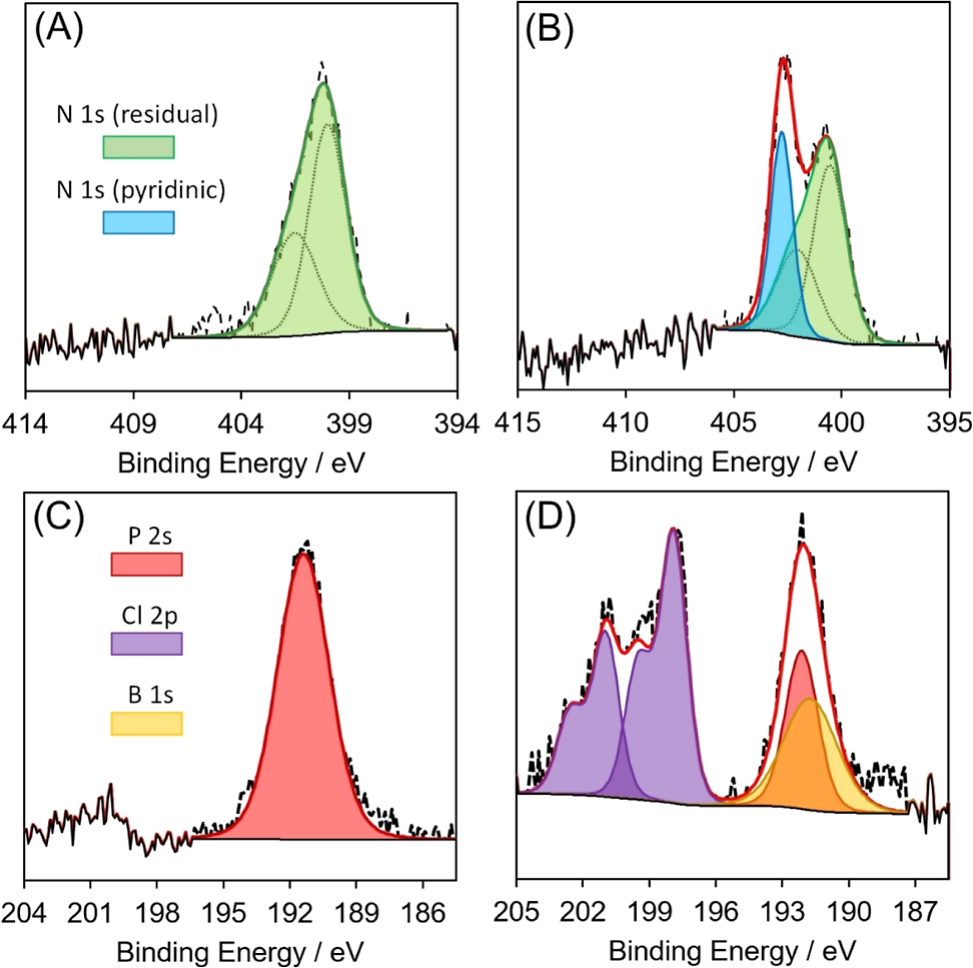
(A) N 1s XP spectra for bare graphene foam, (B) N 1s XP spectra for T1 coated foam, (C) B 1s XP spectra for bare graphene foam, and (D) B 1s XP spectra for T1 coated foam.

Figure 3 shows detailed spectral deconvolutions of the N 1s and B 1s regions of the photoelectron spectroscopy measurements. The dominating elements for both types of samples are C and O (see Table 1) with binding energies consistent with literature reports.^20^ Perhaps surprisingly, the bare graphene foam already contains traces of N (consistent with N-doped carbon at 398.8 eV)^21^ and P. With T1 adsorbed, there are new distinct signals for N 1s at 402 eV [indicative of the pyridinium cation (usually at 401.8 eV)]^22^ and B 1s at 191.6 eV [indicative of the boronic acid (at 191.5 eV)].^23^ Spectral analysis of the B 1s region is complicated by several competing features from the P and Cl contaminants. In order to successfully determine the presence of boron, the peak fits for the P 2s emission at 191 eV were locked to the area intensity of the P 2p emission (since this might result in a 1:1 ratio, and indeed in the bare sample with no boron we do find the anticipated 1:1 ratio). Quantification of the N/B proportion (where N = the pyridinium component only) reveals a ratio of 1.4:1 N/B. Given the relative imprecision in quantification of this boron species due to the phosphorus overlap, this ratio is considered to be within the acceptable range for the anticipated 1:1 ratio of N/B.

**Table 1:** Summary of XPS Data

	C %	O %	B %	P %	N %	Cl %
bare graphene	90.2	8.4	0.0	0.4	1.0	0.0
T1 coated graphene foam	90.1	8.4	0.2	0.1	1.1	0.1

**Figure 4:**
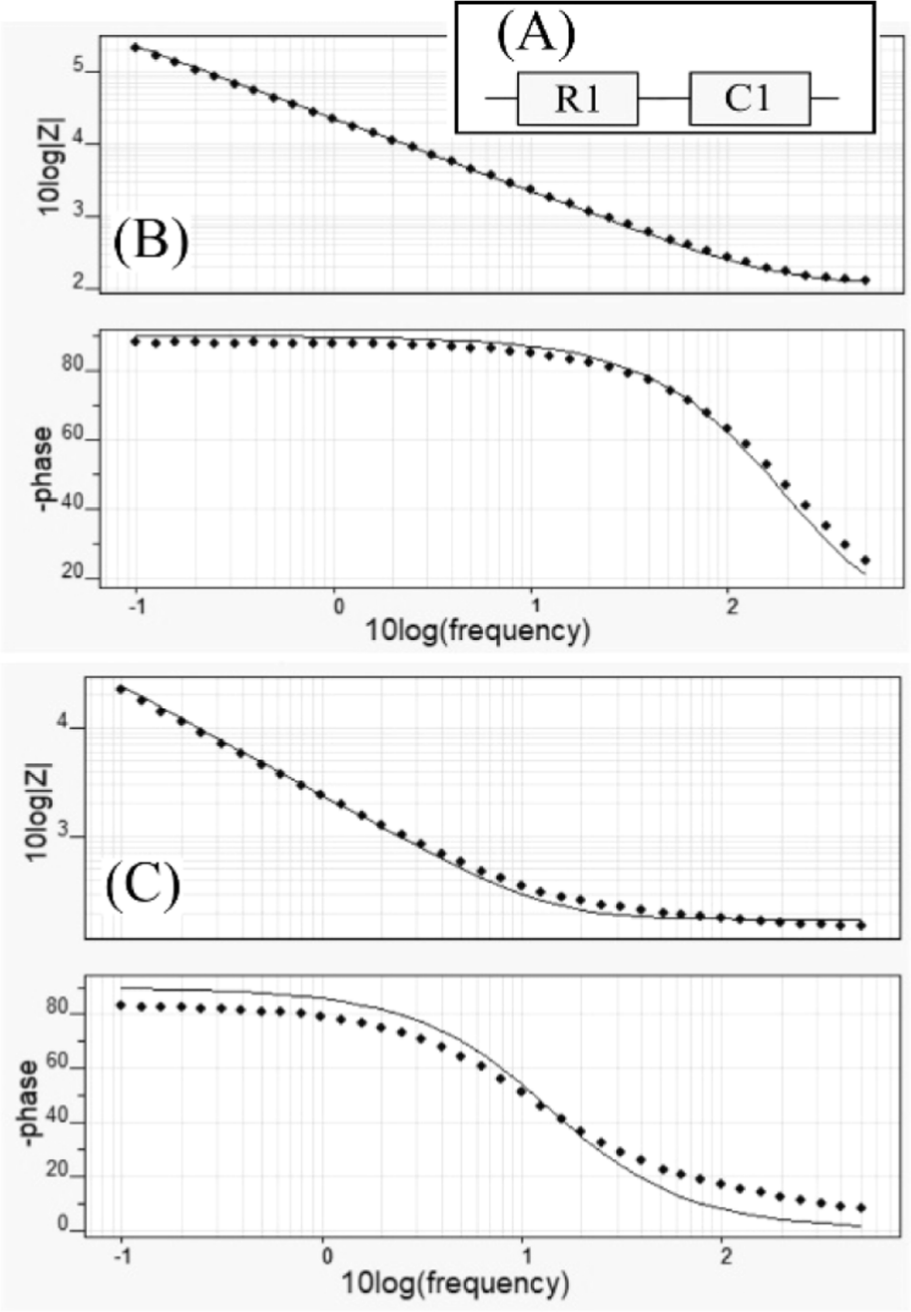
(A) Equivalent circuit used in data analysis. (B) Bode plots of |*Z*| and phase angle versus logarithm of frequency for the bare graphene foam electrode and (C) T1-functionalized (5 μg, drop-cast) graphene foam electrode in 0.1 M phosphate buffer pH 7.

The electrochemical characteristics of bare graphene foam electrodes immersed in an aqueous buffer are dominated by interfacial capacitance and charging. When studying the EIS response at 0.0 V vs Ag/AgCl in 0.1 M phosphate buffer (pH 7) solution, a typical RC-series circuit current response is obtained. Figure 4A shows the equivalent circuit, and Figure 4B summarizes the data in the form of a Bode plot. The line represents the fitting with the equivalent circuit. Table 2 shows the fitted equivalent circuit elements. The series resistance of 118 Ω represents mainly the electrolyte solution component. The capacitance 7.0 μF is associated with the pristine graphene foam. It has been estimated that the roughness factor for these types of electrodes is approximately 100 with a geometric surface area of 12 × 10^–2^ cm^2^.^10^ Therefore, the specific capacitance for graphene foam can be estimated as 0.6 μF cm^–2^, which is low for graphene but consistent with previous reports^24^ (typically for glassy carbon the capacitance is 20 μF cm^–2^ without activation,^25^ and the theoretical/measured limit suggested for graphene in high ionic strength environments^26^ is approximately 21 μF cm^–2^). In previous studies of graphene foam electrode capacitance, similar capacitance values were reported and the effect of surface oxidation on increasing pseudocapacitance was pointed out.^27^ Here, the adsorption of pyrene-appended receptor molecules is investigated.

**Table 2:** Experimental Data for a Bias of 0.0 V; the Graphene Foam Electrode Immersed in 0.1 M Phosphate Buffer Solution pH 7; Results Analysed Using an RC Equivalent Circuit

functionalization	solution	R1/Ohm	% error^a^	C1/μF	% error^a^
bare graphene	pH 7 buffer	118	2.9	7.0	1.1
T1 (5 μg)	pH 7 buffer	116	2.7	66	2.1

a Fitting error based on Ivium fitting software.

When adsorbing the pyrene-appended boronic acid T1 (see eq 1) onto the graphene foam, the resistance R1 is not affected, but the interfacial capacitance is increased by an order of magnitude (Table 2). Figure 3C shows Bode plots with a well-defined RC circuit fit. The amount of T1 (5 μg, 10 nmol or approximately 21 Å^2^ per molecule) has been optimized to represent approximately one monolayer (with slight excess) of the pyrene-appended molecule on the graphene surface.^10^

Functionalization of a bare graphene foam electrode with the pyrene-appended pyridinium boronic acid molecule T1 causes an approximately 10-fold increase in capacitance at the graphene foam electrode surface when compared to the capacitance of the nonfunctionalized electrode. This effect is not pH dependent (investigated from pH 2 to 12) when monitored using EIS at 0.0 V vs Ag/AgCl. Furthermore, the increase in capacitance is not dependent upon applied potential (from −0.4 to +0.4 V vs Ag/AgCl in 0.1 M phosphate buffer pH 7). Therefore, the origin of the capacitance increase must be linked to the molecular structure of the T1 receptor molecule (e.g., the pyridinium charge close to the graphene surface; Figure 1) at the graphene interface and the electronic properties (density of states^28^) of the graphene foam.

To further elucidate the mechanism associated with capacitance increase after T1 adsorption, several structurally comparable molecules including pyrene, pyrene-1-boronic acid, and pyrene-1-carboxaldehyde were adsorbed via drop-casting onto the graphene foam electrode at similar coverage level to that used for T1 adsorption. The capacitance data are summarized in Table 3 (and in Figure S1).

**Table 3:**
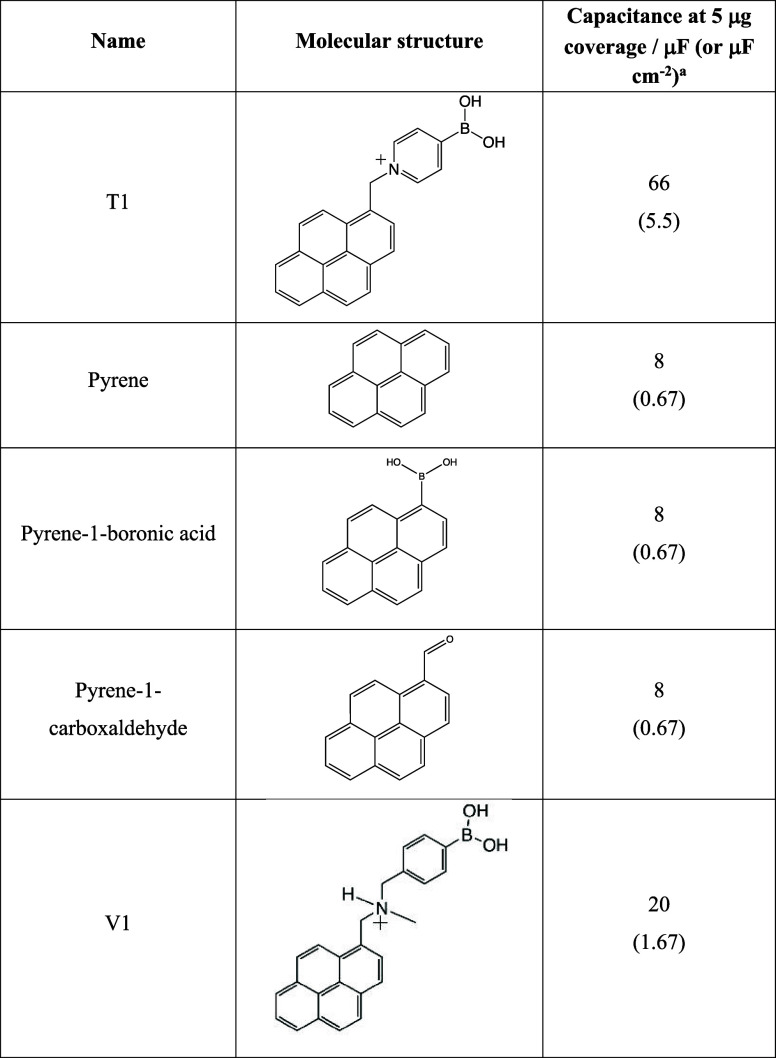
Capacitance Data Collected from Electrochemical Impedance Spectroscopy of Functionalized Graphene Electrodes (Immersed Into 0.1 M Phosphate Buffer Solution; Bias 0.0 V vs. Ag/AgCl) for Several Types of Molecules of Interest at 5 μg per Electrode Surface Coverage

a For a geometric area of approximately 1.2 × 10^–3^ m^2^. Errors estimated at ±10%.

From Table 3, it can be seen that the order of magnitude increase in capacitance upon absorbing T1 to the bare graphene foam electrode appears to be specific to this molecule with a positive pyridinium group close to the pyrene (to attach to the graphene). Therefore, the positively charged pyridinium group present on T1 may contribute to the capacitance increase as this is the only significant structural difference compared with the other molecules. The resultant capacitance increase may be due to the pyridinium interacting with both (i) electronic states within the graphene and (ii) anions in the phosphate buffer electrolyte outside. The anions affect the charge distribution at the interface, which changes the electrical double layer at close distance to the interface and, in this way, potentially increases the capacitance. The structure–function relationship for T1 can be separated into three distinct parts. First, the pyrene group anchors T1 to the graphene surface through strong aromatic π–π interactions. Second, the pyridinium modifies the electrical double layer (and quantum capacitance) via interaction with anions from the bulk electrolyte, yielding an approximately 10-fold increase in capacitance. Third, the boronic acid functional group allows interaction with analyte molecules.

**Figure 5:**
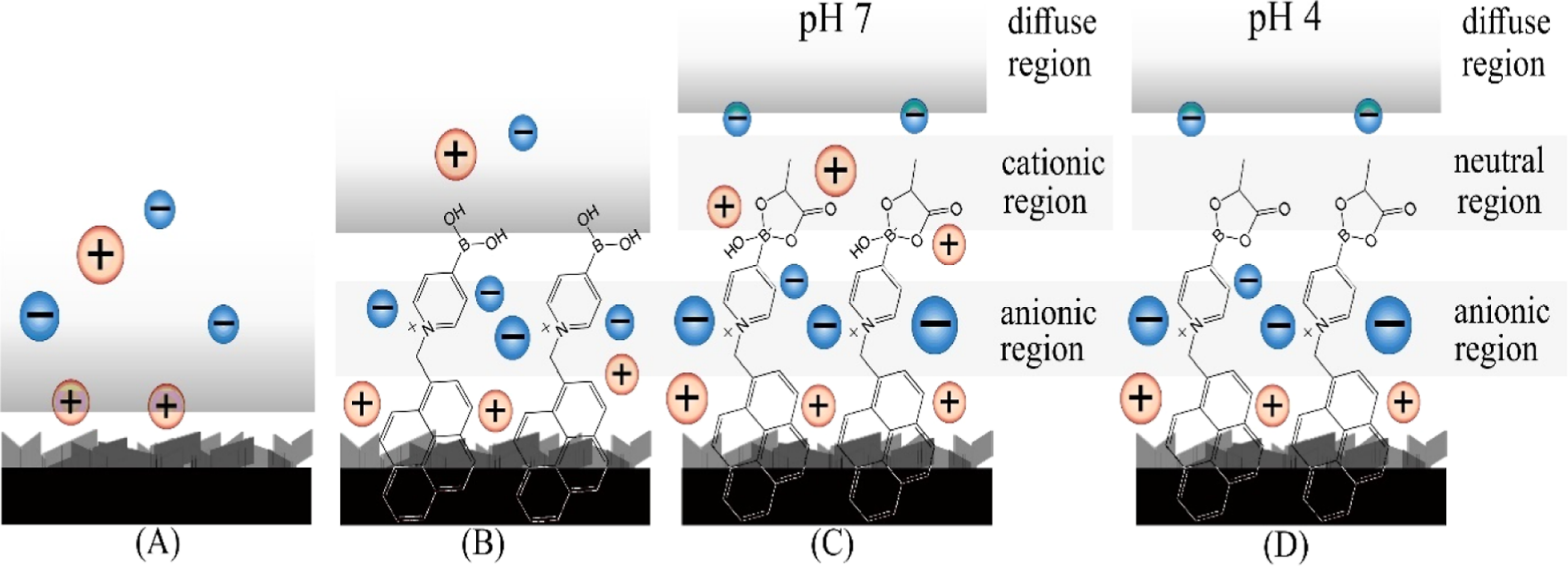
Illustration (hypothetical) of the effect of T1 adsorption (A,B), lactic acid binding (C), and (D) pH on the double layer structure (cations and anions indicated as spheres) at a graphene foam electrode.

The suggested effect of the positive charge in the adsorbed molecule is further confirmed by the capacitance increase observed when another charged pyrene-appended boronic acid (V1, entry 5 in Table 3) is adsorbed onto the graphene foam electrode. This molecule (with a positive charge slightly more remote from the pyrene group) causes an increase in capacitance to approximately 20 μF at 5 μg of coverage. A layer of positive charges is produced at the graphene–solvent interface, and a corresponding layer of negative phosphate anions is likely to be associated. For the case of T1, a graphical illustration (hypothetical) of the interfacial structure is shown in Figure 5. Effects of the positive interfacial charge on the electron density and density of states within the graphene substrate (giving rise to quantum capacitance) are likely.

Capacitance has been suggested as a tool for molecular sensing in work by Bueno et al.,^29^ and the origin of quantum capacitance effects at the nanoscale have been reviewed^30^ and theoretically predicted.^31^ The original observation of quantum capacitance (capacitance associated with the density of electronic states within the bulk material) dates back to work by Gerischer on carbon electrodes.^32^ Capacitance in graphene-based materials has been of high interest in energy storage applications.^33−36^ The edges of graphene surfaces have been suggested to show higher capacitance, and the applied voltage can affect graphene capacitance.^37^ The theoretical limit of graphene capacitance has been estimated as 21 μF cm^–2^^26,38^ (which is higher compared to values reported here), but stacking, impurities, and many other factors contribute. Related phenomena have been reported for graphene materials in contact with ionic liquids.^39^ In the case reported here, a novel effect due to a positively charged adsorbate is suggested. Both the double layer structure and changes in the density of electronic states are likely to contribute to a significant capacitance change. An absorbed positive layer close to the graphene foam surface appears to be important as well as the absence of a balancing negative charge in the same molecule (Figure 5). The effects of the binding of the lactic acid analyte will be considered next.

### Capacitance of Graphene Foam Electrodes: Effects of Lactic Acid

The boronic acid group in T1 acts as a receptor, and it can bind with 1,2-diols^40^ or with α-hydroxy acids such as lactic acid^41^ to form ring structures. Lactic acid binding to the boronic acid receptor may cause further changes in capacitance due to structural changes in the double layer at the graphene foam surface. This effect is monitored using EIS and correlated to the solution analyte concentration, enabling molecular selectivity in detection. Perhaps interestingly, pyrene-1-boronic acid (see Table 3, entry 3) did not show capacitance responses to lactate binding, probably due to not affecting quantum capacitance (vide infra).

Initial tests at pH 7 indicated that there was no further change in capacitance upon binding of lactic acid. However, data in Table 4 (and in Figures S2 and S3) show that at more acidic pH values a clear change happens and the capacitance is increased by lactic acid binding (comparing 0 mM and 100 mM lactic acid in 0.1 M phosphate buffer solution). At pH 4, the capacitance essentially doubles to 111 μF (or 9.2 μF cm^–2^) indicative of a further very significant increase in capacitance. This doubling of capacitance is associated with lactic acid binding and suggests the suitability for further sensor applications.

**Table 4:** Data from Impedance Spectroscopy for Graphene Foam Electrodes Modified with T1 (5 μg) Immersed in Lactate Solutions (0 or 100 mM, in 0.1 M Phosphate Buffer of Identical pH, Volume 100 μL) between pH 3.5 and pH 7.0 (Errors from Triplicate Measurements, Each with a New Electrode)

pH	capacitance with 100 mM lactic acid/μF	% error^a^	capacitance with 0 mM lactic acid/μF	% error^a^	Δcapacitance/μF
3.5	103	2.8	51	3.8	52
4.0	111	3.5	58	3.8	53
4.5	101	3.2	59	3.4	42
5.0	87	2.6	57	3.9	30
5.5	78	2.3	55	3.1	23
6.0	82	2.0	60	3.2	22
6.5	71	1.7	71	2.2	0
7.0	70	1.7	71	2.1	–1

a Error estimate based on Ivium software fitting.

**Figure 6:**
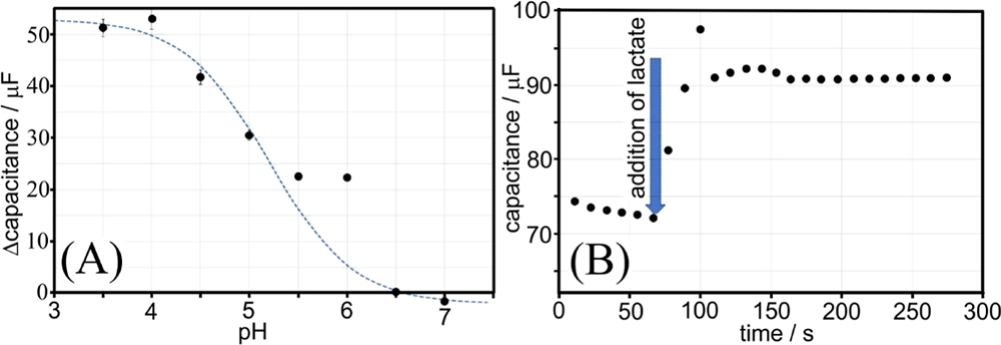
(A) Plot of capacitance change (Δcapacitance) for a T1-functionalized (5 μg) graphene foam electrode immersed in 0.1 M phosphate buffer (from pH 3.5 to pH 7) when going from 0 to 100 mM lactic acid (error bars based on three repeats). The dashed line represents a deprotonation equilibrium for a system with p*K*_A_ = 5.2. (B) Time response of the sensor capacitance at pH 4 with addition of lactate (to give 50 mM lactate) at *t* = 70 s.

Once the sensor electrode has responded to lactic acid, the signal is obtained by EIS and determination of the interfacial capacitance. The sensor does not recover fully (rinsing in pH 7 only partially recovers the signal), and, therefore, a new sensor electrode was employed for each measurement. Figure 6A shows a plot of the change in capacitance (Δcapacitance for going from 0 to 100 mM lactic acid) as a function of pH. At pH 4, a good response of the modified graphene foam electrode to the lactic acid binding is observed. The response curve as a function of pH is not dissimilar (see the fitted dashed line) to a titration curve for a system with p*K*_A_ of 5.2. Boronic acids have a p*K*_A_ in this region (p*K*_A_ of T1 = ∼ 5^10^ and p*K*_A_ of lactic acid = ∼ 3.7^42^), and therefore a hydroxylation equilibrium process can be proposed (eq 1).
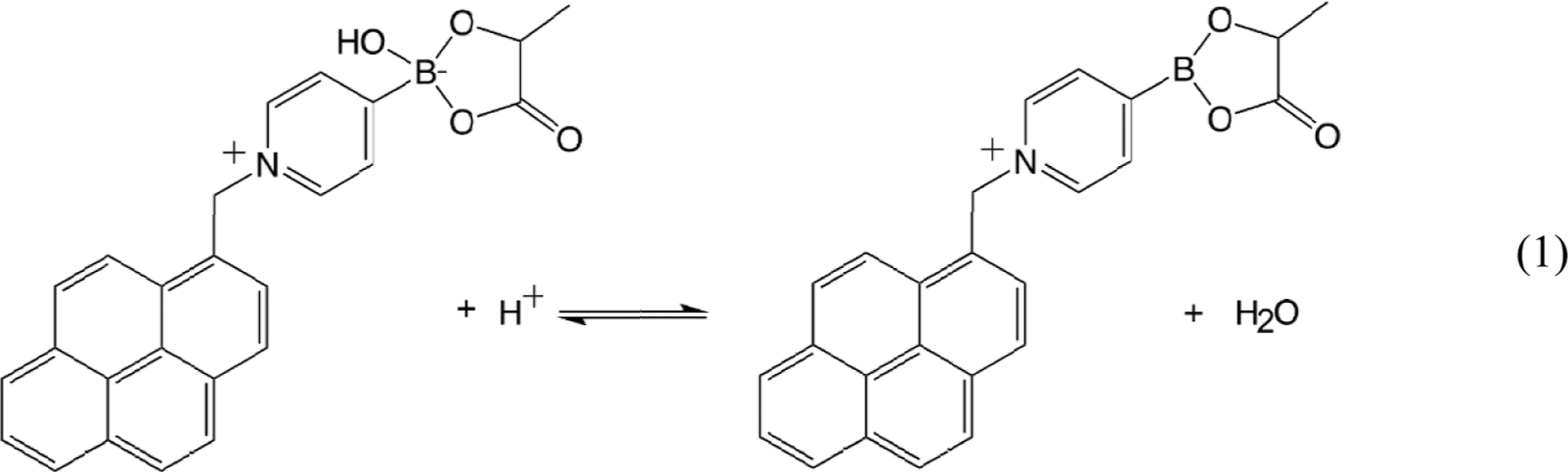
1Further experiments were performed with bare graphene foam electrodes, and no capacitance increase was observed when a 100 mM lactic acid is added at any pH from 3.5 to 7.0. This demonstrates that binding of the T1 receptor to lactic acid is necessary to observe this capacitance doubling effect. The time dependence of the sensor response (Figure 6B) was investigated by rapid repeat impedance measurements in a restricted frequency range (100 to 10 Hz). In pH 4 buffer, the capacitance relaxes slowly to about 72 μF. The addition of lactate (to give 50 mM lactate in pH 4 buffer) causes an instantaneous increase in capacitance (Figure 6B). The value finally settles at about 90 s at 91 μF (after slightly overshooting). The response time is relatively fast and approaches the final value after 20 s.

Figure 5 shows a graphical illustration of the formation of a negatively charged layer in addition to a positively charged layer. The zwitterionic form of T1 is less likely to affect the density of states (quantum capacitance) in the graphene. The cationic form of T1 in conjunction with lactate binding causes the highest change in interfacial capacitance. Perhaps interestingly, in the absence of lactate, the capacitance of the T1-coated graphene foam electrode is not pH dependent, although a hydroxylation equilibrium is anticipated (but probably shifted to lower pH values).

### Impedimetric Lactic Acid Sensing at Graphene Foam Electrodes: Aqueous Buffer Solution

Next, the effect of lactic acid concentration is studied in a pH 4.0 buffer solution. The capacitance increases with increasing concentration of lactic acid (Table 5 and Figure S4), and this can be attributed to binding of lactic acid to the sensor surface. The increase up to 10 mM lactic acid is rapid and close to linear with a further, more gentle increase. Data fitting with a Langmuir isotherm was unsatisfactory (probably due to ionic interactions at the sensor surface or alternatively due to a nonlinear relationship of capacitance and coverage), and therefore the Hill isotherm (eq 2) was selected with one more fitting parameter, α, to describe the effect of interactions.
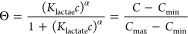
2In this equation, the coverage Θ is linked to the binding constant *K*_A_, the concentration of lactic acid *c*, and the capacitance values going from the limiting values *C*_min_ to *C*_max_. The Hill parameters are *K*_lactate_ = 75 mol^–1^ dm^3^ and α = 0.8. Note that the nonideality (α deviating from 1.0) could be linked to either (A) nonideal effects in solution at the interface causing a real effect on the binding constant or (B) nonideal effects linking the measured capacitance to concentration associated with an apparent effect on the binding constant.

**Table 5:** Data from Electrochemical Impedance Spectroscopy for Graphene Foam Electrodes Modified with T1 (5 μg) Immersed in Lactic Acid Solutions (0–100 mM) in 0.1 M Phosphate Buffer at pH 4.0

lactic acid concentration/mM	capacitance/μF	error/%
0	56.6	3.6
0.1	57.1	3.7
1	65.1	4.1
5	72.7	3.8
10	83.2	3.7
25	86.9	3.9
50	93.5	4.3
100	111	3.9

**Figure 7:**
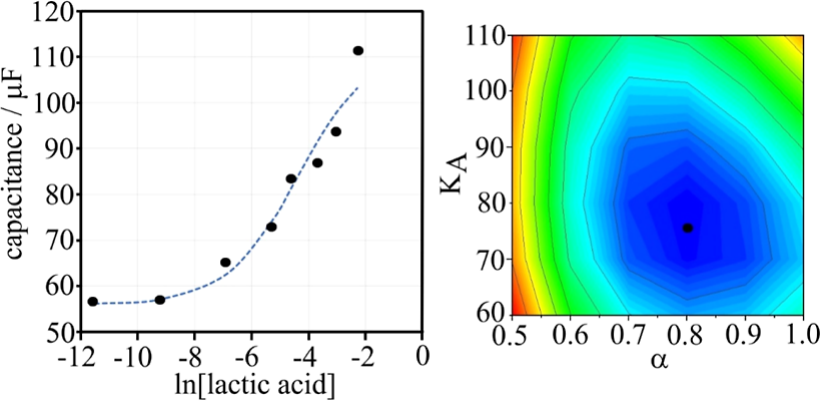
Plot of capacitance versus lactic acid concentration at pH 4.0 for a T1-coated graphene foam electrode in a 0.1 M phosphate buffer. The dashed line corresponds to a Hill isotherm plot. The inset shows the fitting of parameters α and *K*_lactate_.

The linear range for detection of lactic acid in buffer shown in Figure 7 above is approximately 0 to 10 mM lactic acid. Beyond this point, a plateau is observed. Repeat experiments were performed in the 0 to 10 mM lactic acid concentration range in order to determine an effective limit of detection (LoD) value in aqueous phosphate buffer and assess the reproducibility of detection within the critical range (Table 6). The mean LoD value for lactic acid detection in aqueous phosphate buffer is 1.3 mM.

**Table 6:** Data from Impedance Spectroscopy for Graphene Foam Electrodes Modified with T1 (5 μg) Immersed in Lactate Solutions (0–10 mM, Linear Range) at pH 4.0

[LA]/mM	test 1 capacitance/μF	test 2 capacitance/μF	test 3 capacitance/μF
**0**	56.6	53	44.6
**0.1**	57.1	54.6	50.3
**1**	65.1	65.2	64
**5**	72.7	75	81.2
**10**	83.2	85.3	89.8

### Impedimetric Lactic Acid Sensing at Graphene Foam Electrodes: Artificial Sweat

**Figure 8:**
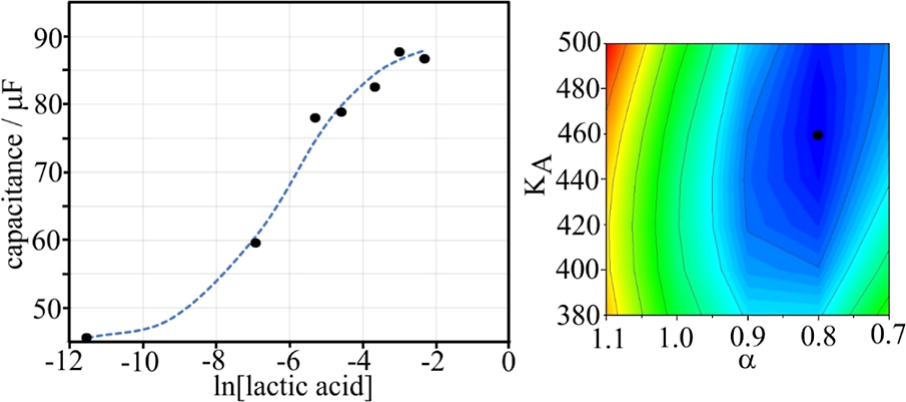
Plot of capacitance versus lactic acid concentration for a T1-coated graphene foam electrode in artificial sweat (pH 4.7). The dashed line corresponds to a Hill isotherm plot. The inset shows the fitting of parameters α and *K*_A_.

An artificial sweat model is employed composed of NaCl, acetic acid, urea, lactic acid, and ammonia in water (see the Experimental Section).^17^ The concentration of lactic acid is varied, and the pH is determined (pH = 4.7). Impedimetric sensing is applied to determine the effects of lactic acid on the electrochemical response. Table 7 (and Figure S5) summarizes the measured capacitance values employing an RC equivalent circuit model. Figure 8 shows a plot of the data. The capacitance increases with lactic acid (with a linear range of 0.1 to 20 mM). A Hill isotherm is employed to fit the data. The binding constant is *K*_A_ = 460 mol^–1^ dm^3^, and the interaction parameter is α = 0.8. The binding constant appears slightly higher when compared with the value in aqueous buffer solution (Table 8).

**Table 7:** Data from Impedance Spectroscopy for Graphene Foam Electrodes Modified with T1 (5 μg) Immersed in Lactate Solutions (0–10 mM, Linear Range) in Artificial Sweat at pH 4.7

[LA]/mM	capacitance/μF
0	45.5
1	59.6
5	77.9
10	78.7
25	82.5
50	87.7
100	86.7

**Table 8:** Data from Impedance Spectroscopy for Graphene Foam Electrodes Modified with T1 (5 μg) Immersed in Artificial Sweat Solutions with Lactate (0–10 mM, Linear Range) at pH 4.7

[LA]/mM	test 1 capacitance/μF	test 2 capacitance/μF	test 3 capacitance/μF
**0**	45.5	43.2	43.8
**1**	59.6	49.2	50.8
**5**	77.9	65.2	64.5
**10**	78.7	78.0	75.7

The linear range for detection of lactic acid in artificial sweat solution shown in Figure 8 is approximately 0.1 to 10 mM lactic acid concentration, with a plateau observed at higher concentrations. A triplicate set of results within the linear range was generated in artificial sweat to produce a limit of detection (LoD) value and assess the reproducibility of this assay in a more complex medium.

From these results, a mean LoD value of 1.4 mM was determined for lactic acid detection in artificial sweat. This compares well to the value in buffer (1.3 mM), showing no interference from the various species in artificial sweat, such as acetic acid, has taken place due to the selectivity of the boronic acid receptor, and lactic acid detection is unaffected. The limit of detection for the graphene foam sensor is comparable to electrochemical lactic acid sensing methods reported in the literature.^43,44^

### Impedimetric Lactic Acid Sensing at Graphene Foam Electrodes: Human Serum

Serum aliquots were spiked with lactic acid (thoroughly vortexed) before adding to the T1-modified electrode for impedimetric analysis at 0.0 V vs Ag/AgCl bias potential. Table 9 (and Figure S6) summarizes the capacitance data obtained by fitting with an RC equivalent circuit.

**Table 9:** Data from Impedance Spectroscopy for Graphene Foam Electrodes Modified with T1 (5 μg) Immersed in Lactate Solutions (0–10 mM, Linear Range) in Human Serum at pH 4.0

[LA]/mM	capacitance/μF
0	54.5
0.1	55.6
1	66.0
5	77.4
10	78.6
25	81.5
50	82.1
100	87.4

**Figure 9:**
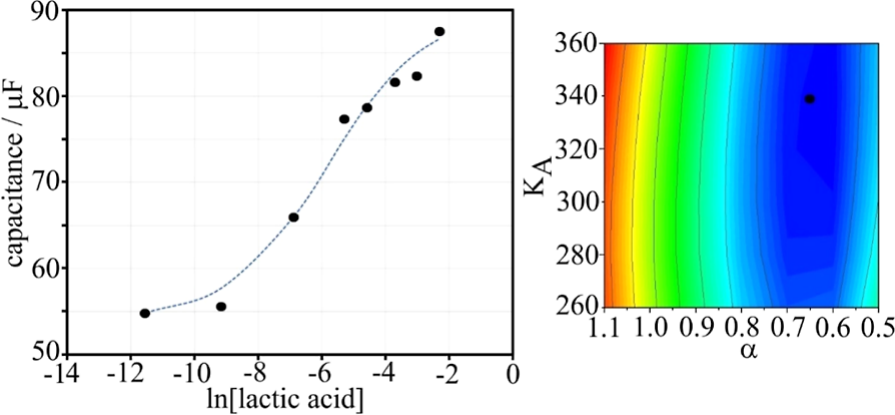
Plot of capacitance versus lactic acid concentration for a T1-coated graphene foam electrode in human serum (pH 4.0). The dashed line corresponds to a Hill isotherm plot. The inset shows the fitting of parameters α and *K*_A_.

Figure 9 shows a plot of capacitance data versus the logarithm of lactic acid concentration. A Hill isotherm was fitted into the data (dashed line) to give *K*_A_ = 340 mol^–1^ dm^3^ and α = 0.65. The binding constant is close to that observed in artificial sweat. The interaction constant is indicative of slightly more deviation from the ideal Langmuir model. The linear range for the detection of lactic acid in serum above is approximated at 0–10 mM, with a plateau clearly observed beyond 10 mM. A triplicate set of results between 0 and 10 mM was again generated in artificial sweat, resulting in a limit of detection (LoD) value (Table 10).

**Table 10:** Data from Impedance Spectroscopy for Graphene Foam Electrodes Modified with T1 (5 μg) Immersed in Serum Solutions Spiked with Lactate (0–10 mM, Linear Range) at pH 4.0

[LA]/mM	test 1 capacitance/μF	test 2 capacitance/μF	test 3 capacitance/μF
**0**	54.5	56.5	51.5
**0.1**	55.6	58.2	52.6
**1**	66.0	67.0	60.1
**5**	77.4	73.8	69.8
**10**	78.7	78.2	76.2

From these results, a mean LoD value of 1.8 mM was determined for lactic acid detection in human serum. Again, this compares favorably to values in buffer (1.3 mM) and artificial sweat (1.4 mM), showing that very little interference from the proteins and other possible interferants has occurred, allowing accurate detection of lactic acid in a clinically relevant range. The accuracy across a range of solutions (sweat/buffer/serum) shows that the capacitance detection methodology using a boronic acid receptor is selective and reproducible, even in biological media.

## Conclusions

It has been shown that the adsorption of a pyrene-appended receptor molecule (T1) onto graphene foam causes significant capacitance changes (when a positively charged pyridinium group is attached close to the graphene surface). The simple modification of the graphene foam electrode with a pyrene-appended boronic acid receptor can provide an effective sensing mechanism based on capacitance changes at the graphene foam electrode surface but only at slightly acidic pH ranges (typically pH 4 to 5). This has been attributed to the p*K*_A_ of the surface attached complex and the need for the formation of a T1-lactate complex with overall positive net charge (to affect the quantum capacitance). Future development of modified receptor molecules could help improve the reversibility of lactate binding and shift the optimum sensor pH further into neutral pH (for in vivo sensing at pH 6 to 7).

The ability to enhance interfacial capacitance by the adsorption of charged molecules onto graphene foam is intriguing and possibly important in a wider range of contexts. The mechanism for the change in interfacial capacitance has been attributed here to a combination of (i) interfacial structure and charge distribution and (ii) associated changes in the density of states within the graphene foam substrate (i.e., quantum capacitance). The change in capacitance upon adsorption is of interest, and further study is required to further push the limits of graphene foam capacitance. The additional change in capacitance upon molecular recognition and binding of lactate allows molecular recognition and sensing even though capacitance used to be a nonselective sensing tool. The molecular selectivity of boronic acid is coupled to the capacitance change at the interface.

In the future, the question of sensor recovery and sensor reuse needs to be addressed. A wider range of boronic acid receptor molecules should be developed and screened to provide deeper insight into interfacial processes, including (i) the mode of surface attachment and (ii) the kinetics of analyte binding and unbinding. The use of quantum capacitance in sensing opens up new opportunities in wireless sensing. Opportunities based on molecular “quantum capacitance enhancers” need to be investigated in more depth.

## Supporting Information Available

Bode plots of |Z| and phase angle *versus* logarithm of frequency for a graphene foam electrode coated with different molecules at pH 7, including molecule T1 immersed in buffer solution at different pH and different lactic acid concentrations, T1 immersed in artificial sweat solution at pH4.7 with different lactic acid concentrations, and T1 immersed in serum at pH 4.0 with different lactic acid concentrations, and details of synthesis and characterization of molecule V1 (PDF)
