# Supplementary material for: Pyrene-Appended Boronic Acids on Graphene Foam Electrodes Provide Quantum Capacitance-Based Molecular Sensors for Lactate
Source: ACS Sens. 2024 Mar 6;9(3):1565–74. doi: 10.1021/acssensors.4c00027 (PMC10964244; doi:10.1021/acssensors.4c00027)
Supplement: Supplementary file 1 — se4c00027_si_001.pdf [file se4c00027_si_001.pdf]

Supporting Information

Pyrene-Appended Boronic Acids on Graphene Foam Electrodes Provide Quantum Capacitance-Based Molecular Sensors for Lactate

---

Simon M. Wikeley [1], Jakub Przybylowski [1], Jordan E. Gardiner [1], Tony D. James [1,2], Philip J. Fletcher [3], Mark A. Isaacs [4,5], Pablo Lozano-Sanchez [6], Marco Caffio [6], and Frank Marken*[1]

[1] Department of Chemistry, University of Bath, Claverton Down, Bath BA2 7AY, UK
[2] School of Chemistry and Chemical Engineering, Henan Normal University, Xinxiang 453007, China.
[3] University of Bath, Imaging Facility, Bath BA2 7AY, UK
[4] HarwellXPS, Research Complex at Harwell, STFC Rutherford Appleton Laboratory, Harwell Campus, Didcot, OX11 0FA, UK
[5] Department of Chemistry, University College London, London, WC1H 0AJ
[6] Integrated Graphene Ltd., Euro House, Wellgreen Place, Stirling FK8 2DJ, UK

Contents

Figure S1. (A-D) Bode plots of |Z| and phase angle *versus* logarithm of frequency for a graphene foam electrode coated with 5 μg of several molecules of interest named (A) pyreneboronic acid (B) pyrenecarboxaldehyde and (C) pyrene immersed in 0.1 M phosphate buffer at pH 7. (D) Equivalent circuit used in data analysis. **S3**

Figure S2. (A-H) Bode plots of |Z| and phase angle *versus* logarithm of frequency for a graphene foam electrode coated with 5ug of T1 immersed in 0.1M phosphate buffer at varying pH (A) pH 3.5 (E_{bias} = 0.0 V vs. Ag/AgCl), (B) pH 4.0 (E_{bias} = 0.0 V vs. Ag/AgCl), (C) pH 4.5 (E_{bias} = 0.0 V vs. Ag/AgCl), (D) pH 5.0 (E_{bias} = 0.0 V vs. Ag/AgCl), (E) pH 5.5 (E_{bias} = 0.0 V vs. Ag/AgCl), (F) pH 6.0 (E_{bias} = 0.0 V vs. Ag/AgCl), (G) pH 6.5 (E_{bias} = 0.0 V vs. Ag/AgCl) and (H) pH 7.0 (E_{bias} = 0.0 V vs. Ag/AgCl). (I) Equivalent circuit used in data analysis. **S4**

Figure S3. (A-H) Bode plots of |Z| and phase angle *versus* logarithm of frequency for a graphene foam electrode coated with 5ug of T1 immersed in a solution of 0.1M phosphate buffer spiked with 100m M lactic acid at varying pH (A) pH 3.5 (E_{bias} = 0.0 V vs. Ag/AgCl), (B) pH 4.0 (E_{bias} = 0.0 V vs. Ag/AgCl), (C) pH 4.5 (E_{bias} = 0.0 V vs. Ag/AgCl), (D) pH 5.0 (E_{bias} = 0.0 V vs. Ag/AgCl), (E) pH 5.5 (E_{bias} = 0.0 V vs. Ag/AgCl), (F) pH 6.0 (E_{bias} = 0.0 V vs. Ag/AgCl), (G) pH 6.5 (E_{bias} = 0.0 V vs. Ag/AgCl) and (H) pH 7.0 (E_{bias} = 0.0 V vs. Ag/AgCl). (I) Equivalent circuit used in data analysis. **S5**

Figure S4. (A-H) Bode plots of |Z| and phase angle *versus* logarithm of frequency for a graphene foam electrode coated with 5ug of T1 immersed in a solution of 0.1M phosphate buffer at pH 4.0 vs Ag/AgCl containing varying concentrations of lactic acid (A) 0 mM (B) 0.1mM, (C) 1mM, (D) 5 mM, (E) 10 mM, (F) 25 mM, (G) 50 mM, (H) 100 mM lactic acid. (I) Equivalent circuit used in data analysis. **S6**

Figure S5. (A-G) Bode plots of |Z| and phase angle *versus* logarithm of frequency for a graphene foam electrode coated with 5ug of T1 immersed in artificial sweat solution at pH 4.7 vs Ag/AgCl containing varying concentrations of lactic acid (A) 0 mM (B) 1mM, (C) 5 mM, (D) 10 mM, (E) 25 mM, (F) 50 mM, (G) 100 mM lactic acid. (H) Equivalent circuit used in data analysis. **S7**

Figure S6. (A-H) Bode plots of |Z| and phase angle *versus* logarithm of frequency for a graphene foam electrode coated with 5ug of T1 immersed in serum at pH 4.0 vs Ag/AgCl spiked with varying concentrations of lactic acid (A) 0 mM (B) 0.1mM, (C) 1mM, (D) 5 mM, (E) 10 mM, (F) 25 mM, (G) 50 mM, (H) 100 mM lactic acid. (I) Equivalent circuit used in data analysis. **S8**

Synthesis and Characterisation of N-methyl-1-(pyren-1-yl)-N-(4-(4,4,5,5-tetramethyl-1,3,2-dioxaborolan-2-yl)benzyl)methanamine (V1). **S9**

Figure S1. (A-D) Bode plots of |Z| and phase angle *versus* logarithm of frequency for a graphene foam electrode coated with 5 μg of several molecules of interest named (A) pyreneboronic acid (B) pyrenecarboxaldehyde and (C) pyrene immersed in 0.1 M phosphate buffer at pH 7. (D) Equivalent circuit used in data analysis.

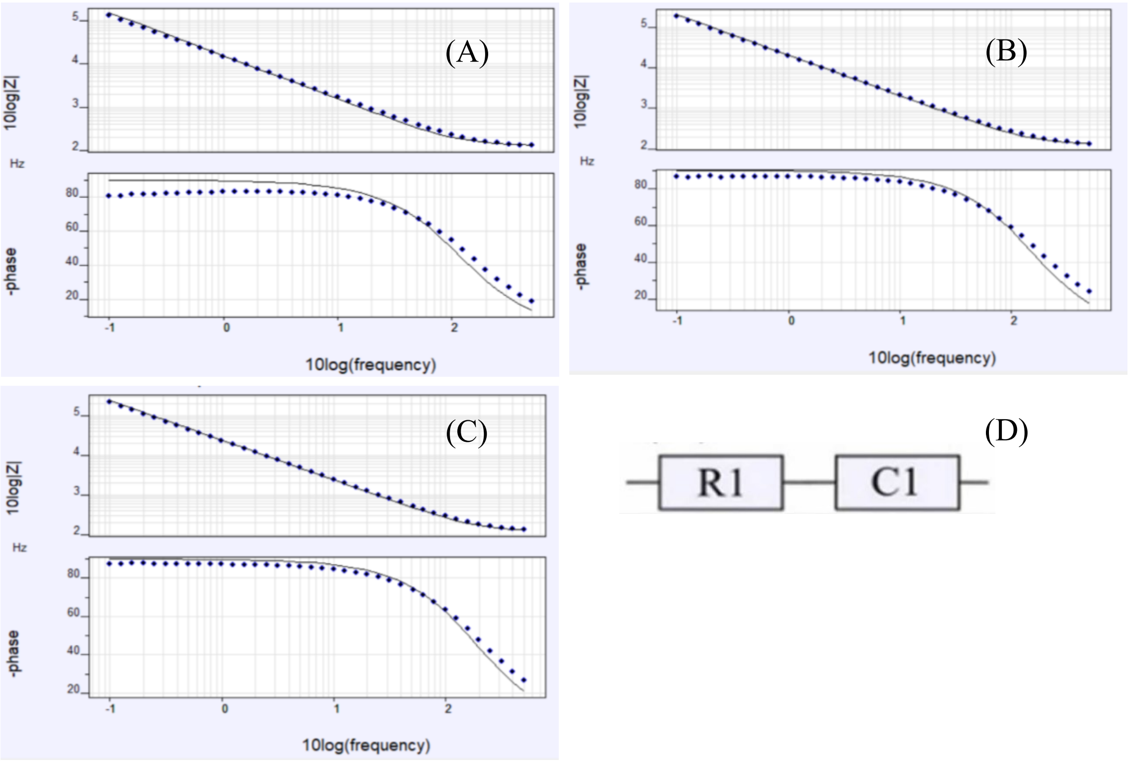

Figure S2. (A-H) Bode plots of |Z| and phase angle *versus* logarithm of frequency for a graphene foam electrode coated with 5 μg of T1 immersed in 0.1 M phosphate buffer at varying pH (A) pH 3.5 (E_{bias} = 0.0 V vs. Ag/AgCl), (B) pH 4.0 (E_{bias} = 0.0 V vs. Ag/AgCl), (C) pH 4.5 (E_{bias} = 0.0 V vs. Ag/AgCl), (D) pH 5.0 (E_{bias} = 0.0 V vs. Ag/AgCl), (E) pH 5.5 (E_{bias} = 0.0 V vs. Ag/AgCl), (F) pH 6.0 (E_{bias} = 0.0 V vs. Ag/AgCl), (G) pH 6.5 (E_{bias} = 0.0 V vs. Ag/AgCl) and (H) pH 7.0 (E_{bias} = 0.0 V vs. Ag/AgCl). (I) Equivalent circuit used in data analysis.

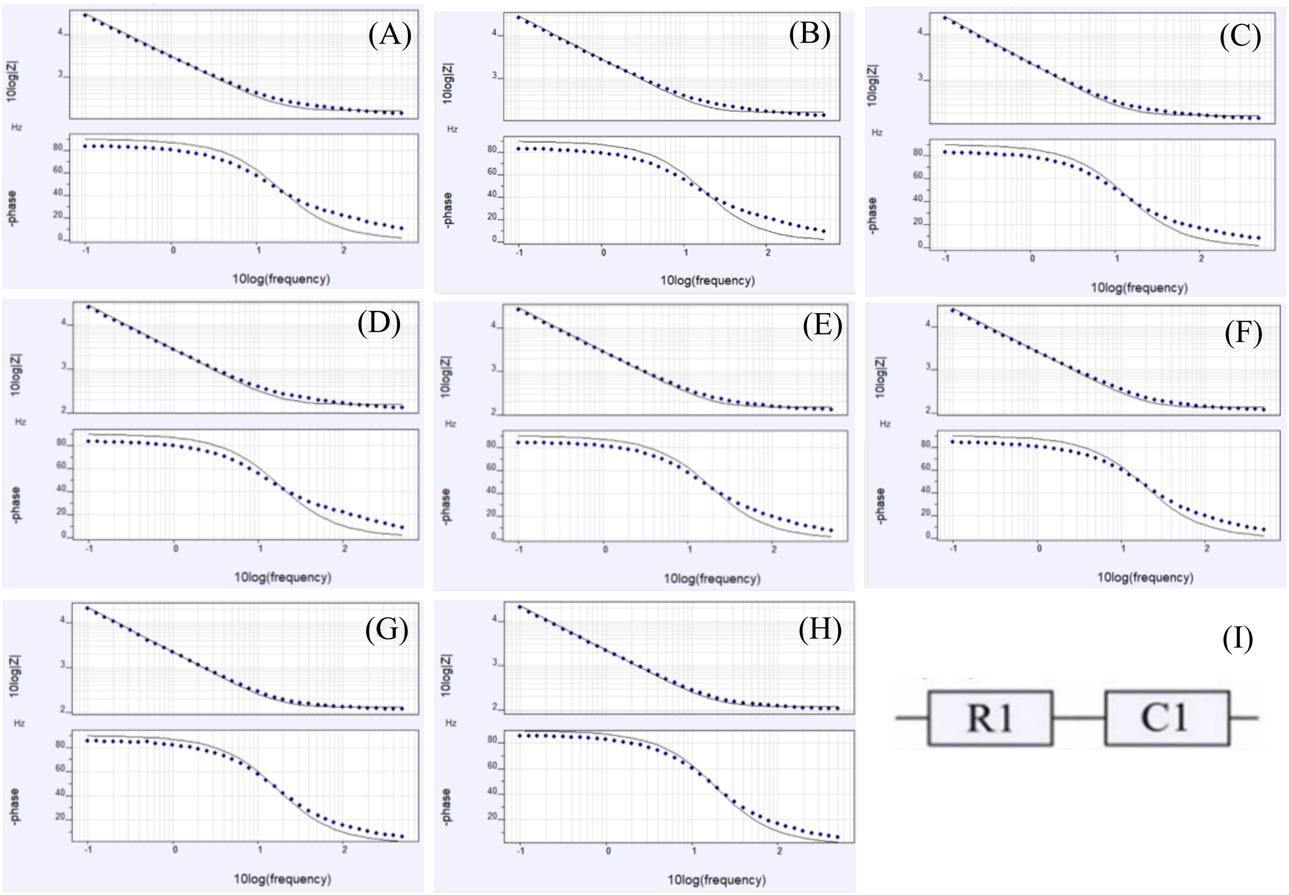

Figure S3. (A-H) Bode plots of |Z| and phase angle *versus* logarithm of frequency for a graphene foam electrode coated with 5 μg of T1 immersed in a solution of 0.1 M phosphate buffer spiked with 100 mM lactic acid at varying pH (A) pH 3.5 (E_{bias} = 0.0 V vs. Ag/AgCl), (B) pH 4.0 (E_{bias} = 0.0 V vs. Ag/AgCl), (C) pH 4.5 (E_{bias} = 0.0 V vs. Ag/AgCl), (D) pH 5.0 (E_{bias} = 0.0 V vs. Ag/AgCl), (E) pH 5.5 (E_{bias} = 0.0 V vs. Ag/AgCl), (F) pH 6.0 (E_{bias} = 0.0 V vs. Ag/AgCl), (G) pH 6.5 (E_{bias} = 0.0 V vs. Ag/AgCl) and (H) pH 7.0 (E_{bias} = 0.0 V vs. Ag/AgCl). (I) Equivalent circuit used in data analysis.

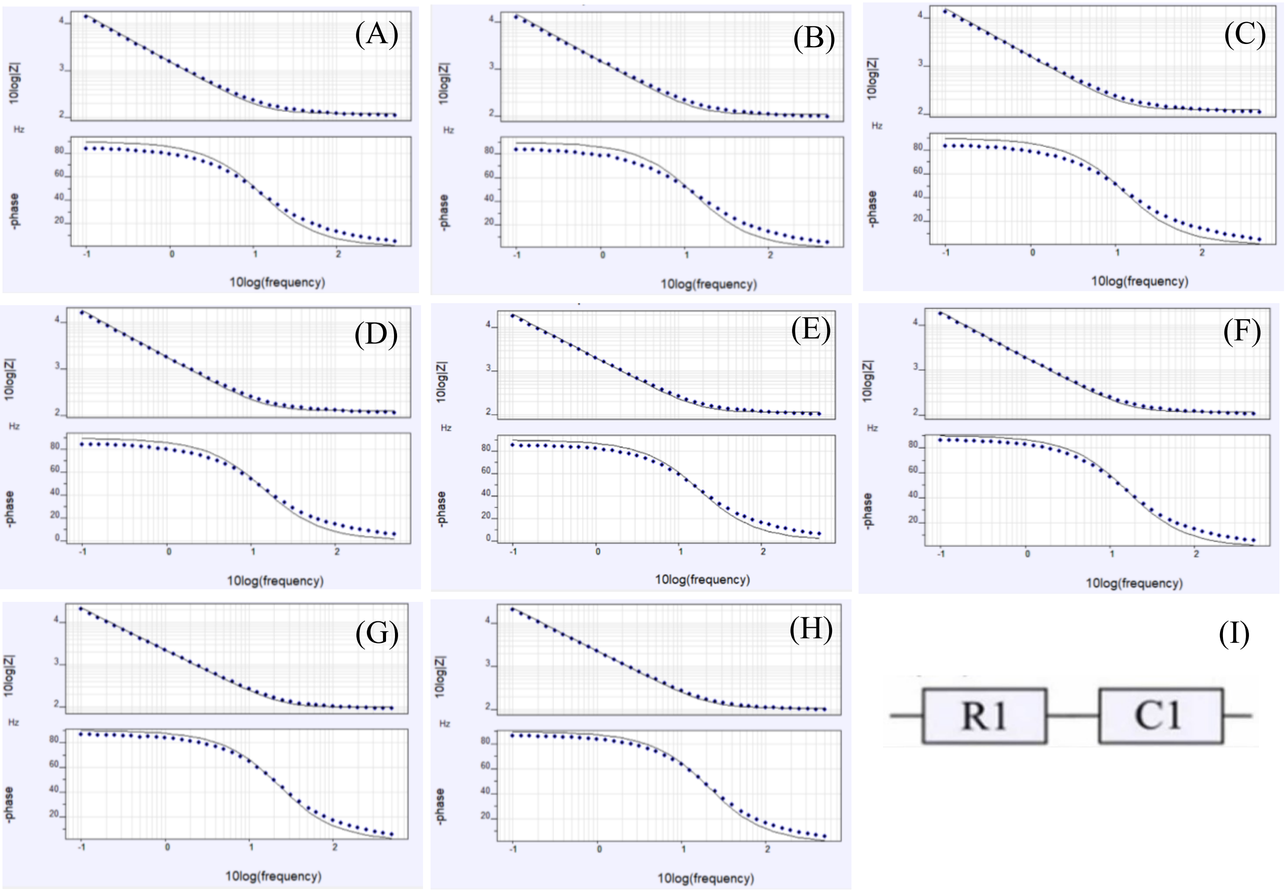

Figure S4. (A-H) Bode plots of |Z| and phase angle *versus* logarithm of frequency for a graphene foam electrode coated with 5 μg of T1 immersed in a solution of 0.1 M phosphate buffer at pH 4.0 vs Ag/AgCl containing varying concentrations of lactic acid (A) 0 mM (B) 0.1 mM, (C) 1 mM, (D) 5 mM, (E) 10 mM, (F) 25 mM, (G) 50 mM, (H) 100 mM lactic acid. (I) Equivalent circuit used in data analysis.

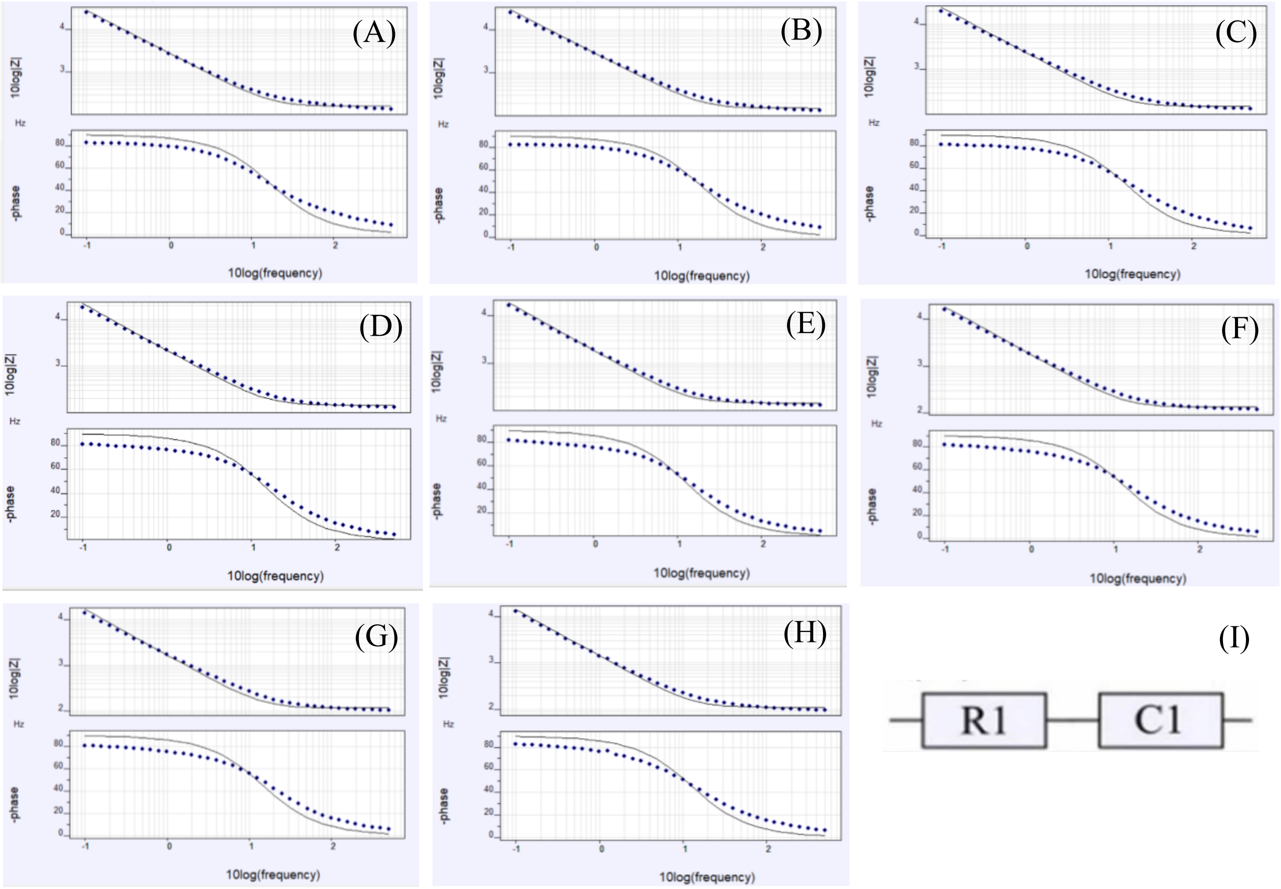

Figure S5. (A-G) Bode plots of |Z| and phase angle *versus* logarithm of frequency for a graphene foam electrode coated with 5 µg of T1 immersed in artificial sweat solution at pH 4.7 vs Ag/AgCl containing varying concentrations of lactic acid (A) 0 mM (B) 1mM, (C) 5 mM, (D) 10 mM, (E) 25 mM, (F) 50 mM, (G) 100 mM lactic acid. (H) Equivalent circuit used in data analysis.

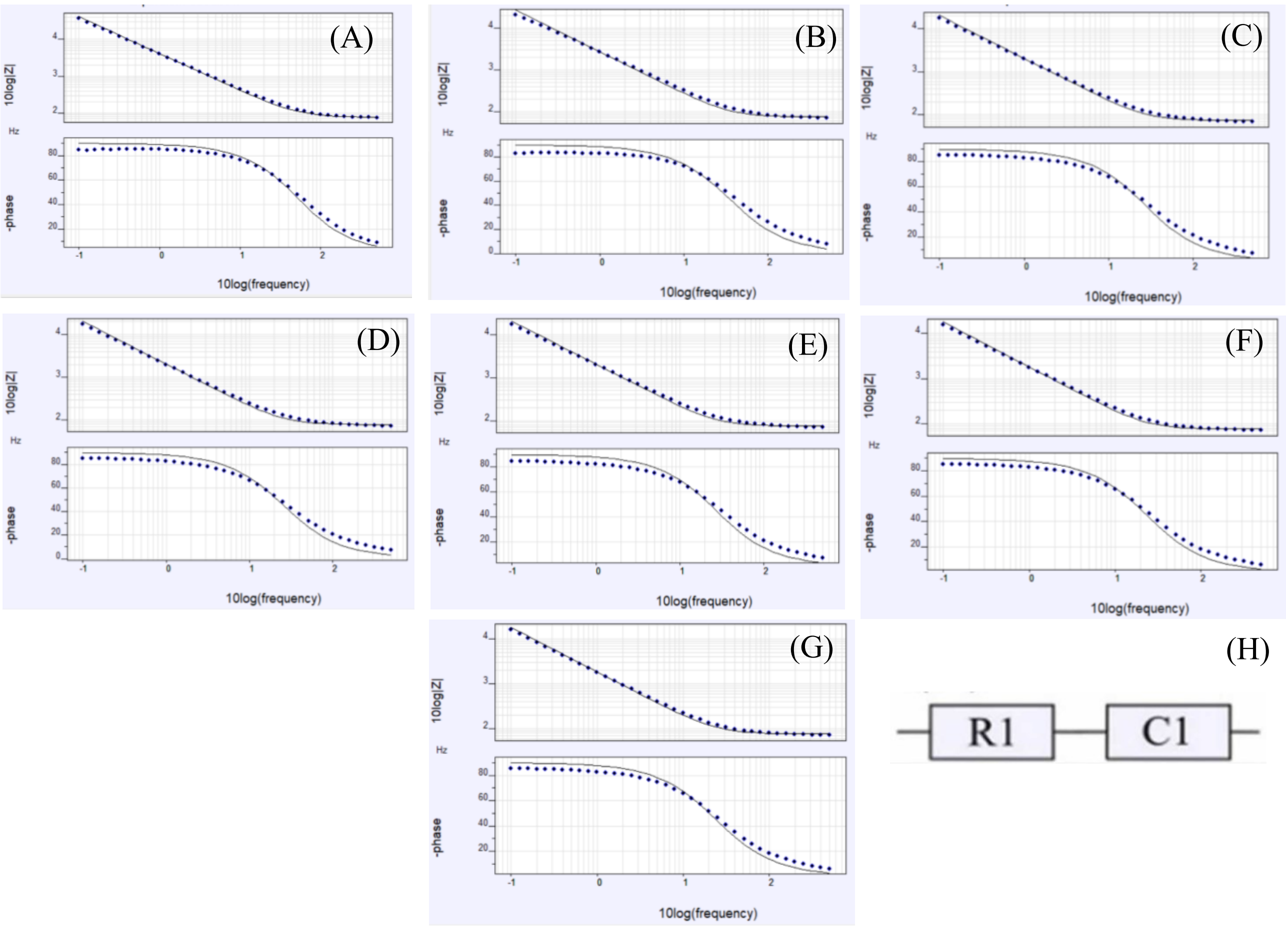

Figure S6. (A-H) Bode plots of |Z| and phase angle *versus* logarithm of frequency for a graphene foam electrode coated with 5 µg of T1 immersed in serum at pH 4.0 vs Ag/AgCl spiked with varying concentrations of lactic acid (A) 0 mM (B) 0.1mM, (C) 1mM, (D) 5 mM, (E) 10 mM, (F) 25 mM, (G) 50 mM, (H) 100 mM lactic acid. (I) Equivalent circuit used in data analysis.

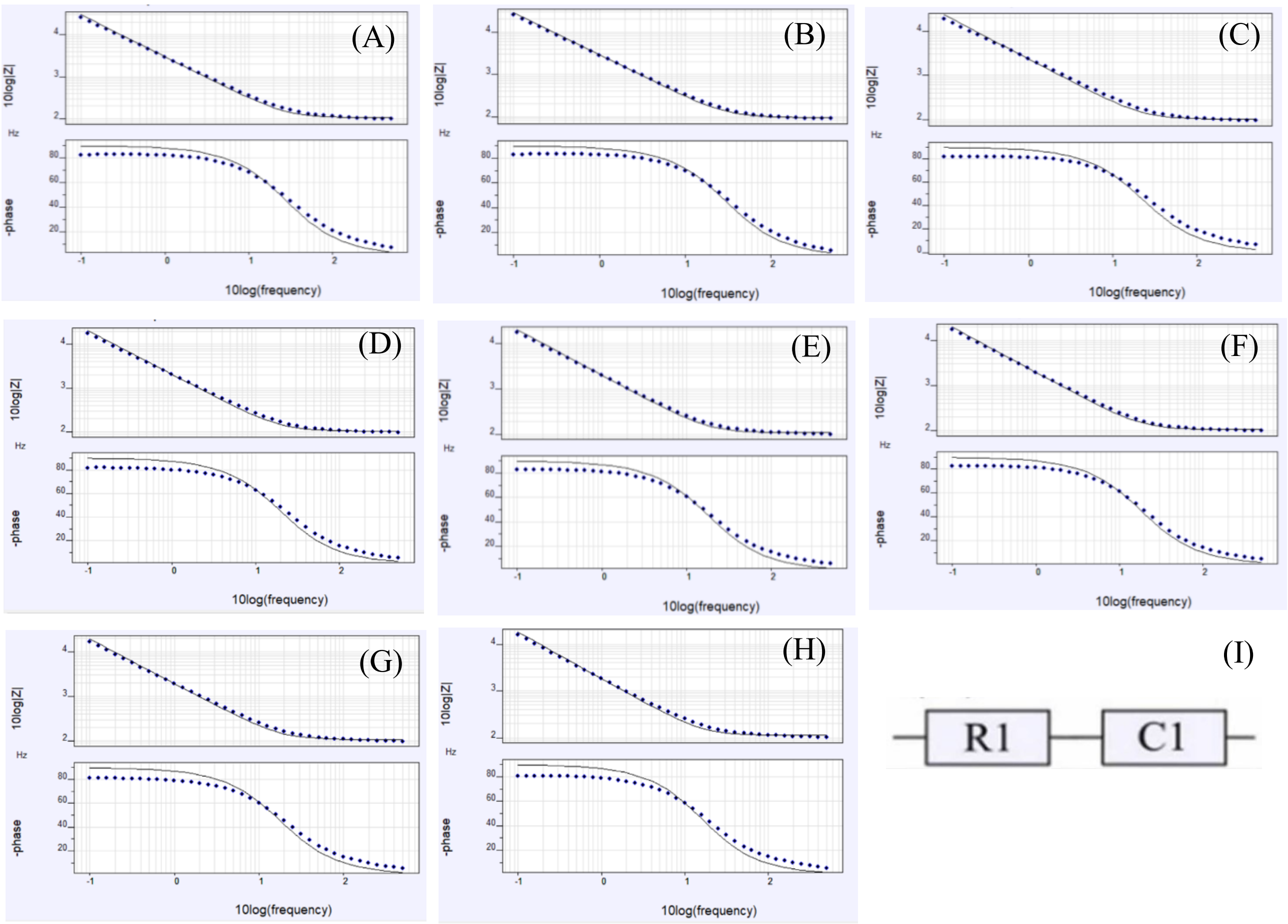

Synthesis and Characterisation of N-methyl-1-(pyren-1-yl)-N-(4-(4,4,5,5-tetramethyl-1,3,2-dioxaborolan-2-yl)benzyl)methanamine (V1)

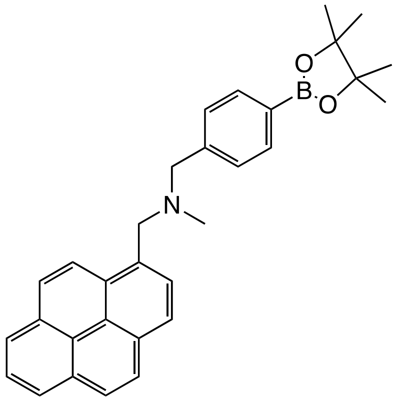

N-methyl-1-(pyren-1-yl)-*N*-(4-(4,4,5,5-tetramethyl-1,3,2-dioxaborolan-2-yl)benzyl)methanamine; Chemical Formula: $C_{31}H_{32}BNO_2$.

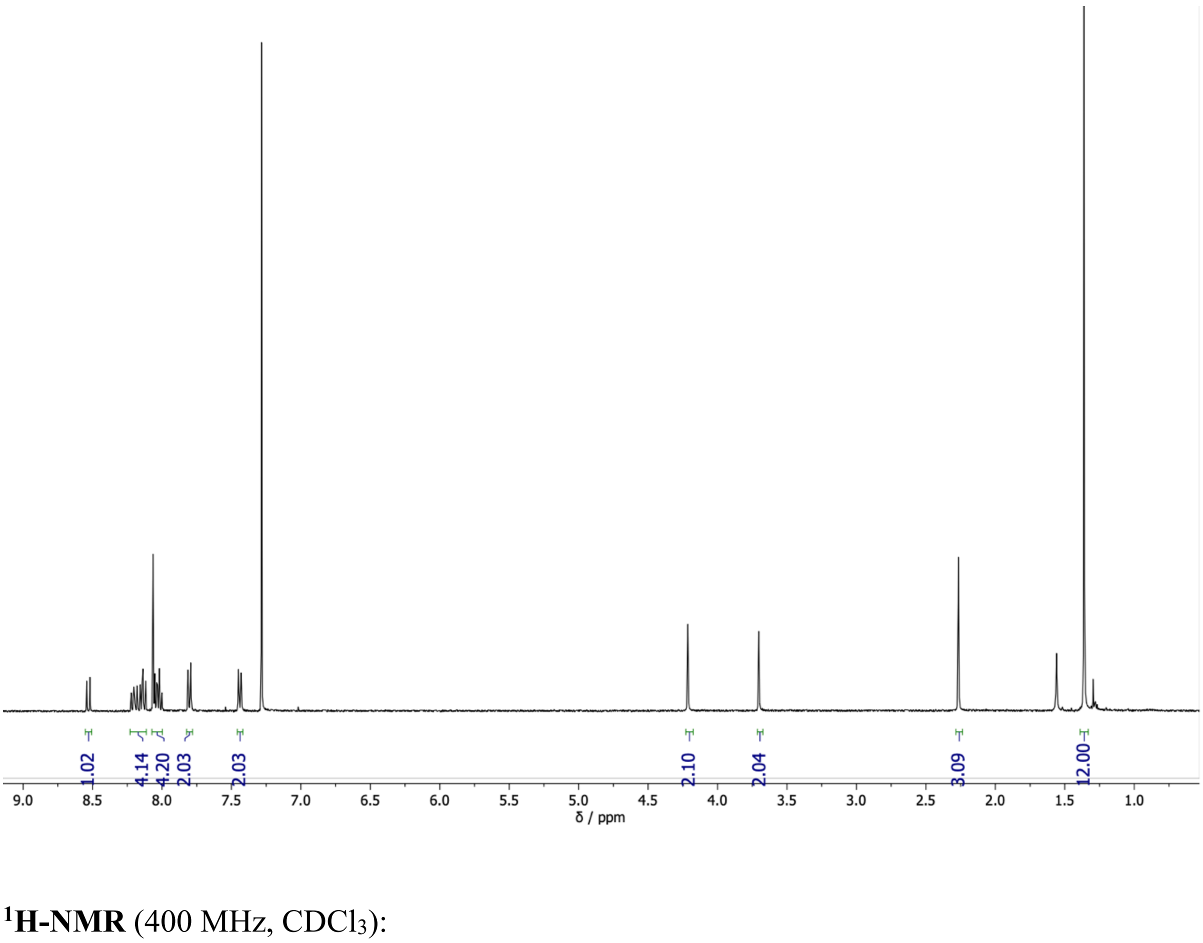

^{1}H-NMR (400 MHz, $CDCl_3$):

δ 8.6 - 8.2 (m, 9H, pyr-H), δ 7.8 (d, 2H, Ar-H), δ 7.5 (d, 2H, Ar-H), δ 4.2 (s, 2H, N-CH_2), δ 3.7 (s, 2H, N-CH_2), δ 2.3 (s, 3H, N-CH_3), δ 1.3 (s, 12H, Bpin).

Figure: Extracted ion chromatogram (EIC) of compound.

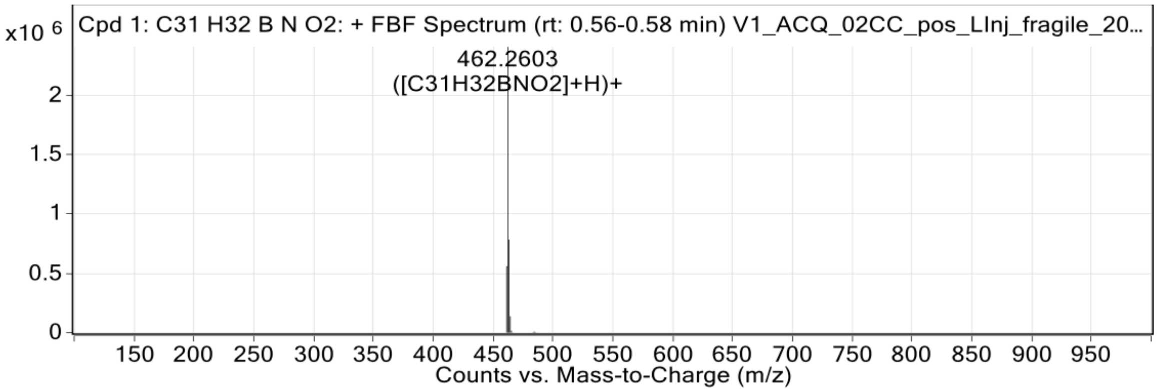

Figure: Full range view of Compound spectra and potential adducts.

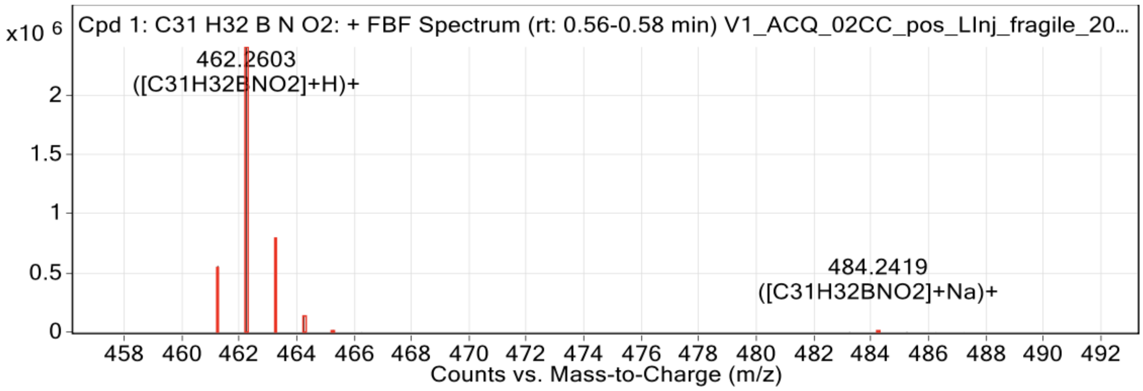

HRMS: (ESI): m/z calculated for $C_{31}H_{32}BNO_2$: 462.2599 for $[M+H]^+$, found 462.2603.

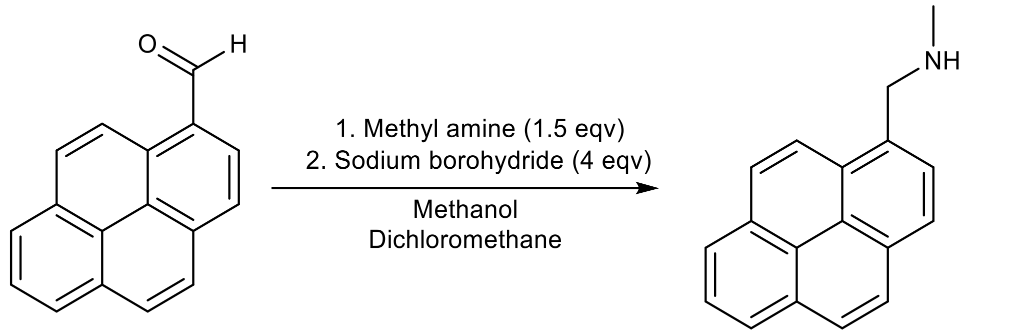

1-pyrenecarboxaldehyde
(1 eqv)

N-methyl-1-(pyren-1-yl)methanamine

Scheme A. Reductive amination of pyrenecarboxaldehyde to form the first intermediate for V1.

Pyrene carboxaldehyde (5.00 g, 21.72 mmol) was dissolved in methanol (100 mL) and dichloromethane (100 mL). To the dissolved aldehyde sodium sulphate (3 g) was added. 2M methylamine in methanol (22.00 mL, 43.43 mmol) was added slowly to the solution, and it was left to stir at room temperature for 4 hours. At 0ºC sodium borohydride (3.29 g, 86.86 mmol) was added slowly to the solution and left to stir at 0ºC for a further hour. 1 M hydrochloric acid (25 mL) was used to quench the reaction at 0ºC. The excess solvent was removed, and the crude was extracted with sodium bicarbonate (40 mL). The aqueous layer was extracted with dichloromethane (3 x 50 mL), washed with water (2 x 100 mL) and brine (100). The organic layer was dried with magnesium sulphate and the excess solvent was removed under reduced pressure. The obtained product was a viscous yellow oil that solidifies over time (5.23 g, 89%)

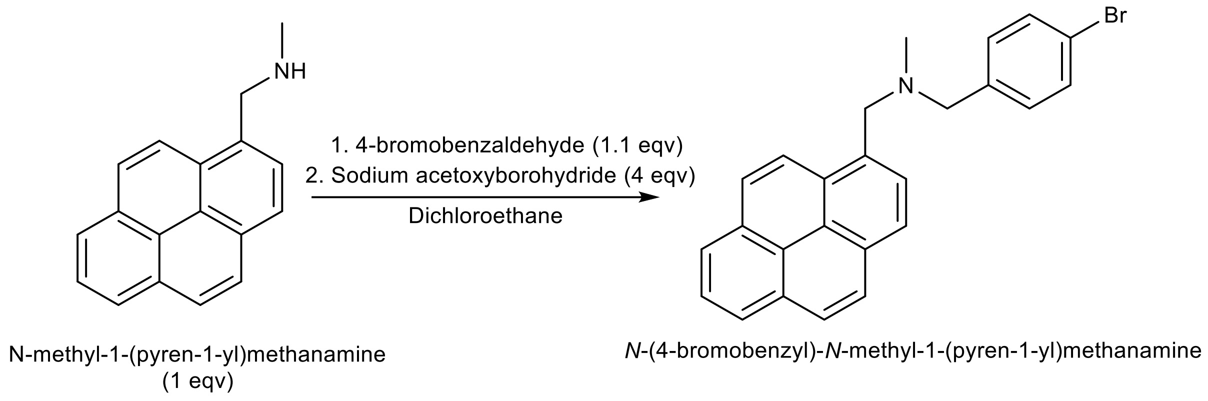

Scheme B. Reductive amination to obtain the second intermediate for V1

The amine (0.84 g, 3.42 mmol) and the starting aldehyde (0.565 g, 3.77 mmol) were left to stir in dry dichloroethane (50 mL) with molecular sieves (1 g) for 4 hours. Sodium acetoxy borohydride was added to the stirring solution at 0ºC. The reaction was left to stir at 0ºC for an hour, then a further 2 hours at room temperature. The reaction was quenched with sodium bicarbonate (15 mL) until pH 7/8. The aqueous layer was extracted with dichloromethane (2 x 50 mL) then the combined organic layers were washed with water (3 x 100 mL) and brine (100 ml). The organic layer was dried with magnesium sulphate and excess solvent was removed under reduced pressure. The crude was further purified by flash column chromatography using 70% ethyl acetate and 30% petroleum ether eluent system to obtain pure product (1.42 g, 88%).

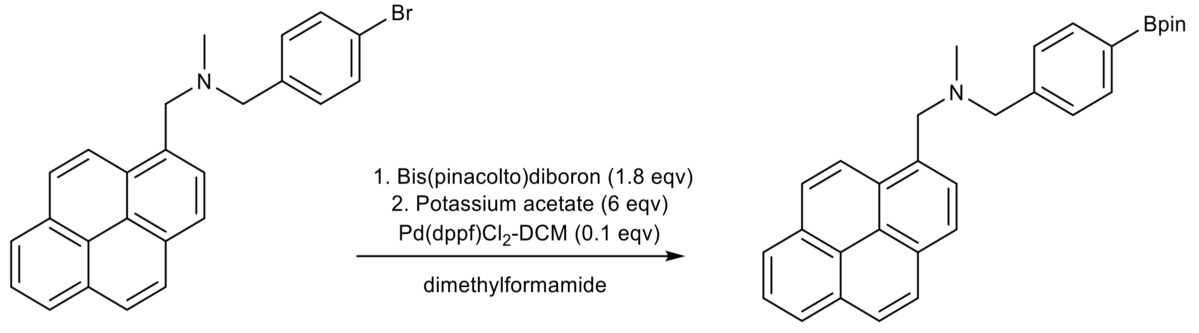

N-(2-bromobenzyl)-N-methyl-1
-(pyren-1-yl)methanamine

Scheme C. Suzuki-Miyaura borylation to obtain V1 protected with pinacol.

Bis(pinacolto)diboron (1.36 g, 5.34 mmol), potassium acetate (0.86 g, 17.8 mmol), starting bromide (1.23 g, 2.96 mmol) and the Pd catalyst (0.242 g, 0.30 mmol) were all placed under an inert atmosphere, followed by addition of dry dimethylformamide (25ml). Nitrogen was further bubbled through the solution. The reaction was heated up to 95ºC and left to stir for16 hours. Once the reaction reached completion it was cooled and water (200 mL) with ethyl acetate (200 mL) was added to the reaction mixture. The layers were separated, and aqueous layer was extracted with ethyl acetate (2 x 100 mL) then washed with water (5 x 200 mL) and brine (5 x 100 mL). The organic layer was dried with $MgSO_4$ and excess solvent was removed under reduced pressure to give the crude product. The crude product was dissolved in diethyl ether and gently heated, followed by a filtration with a cotton plug. The compound was then further purified via reverse-phase column chromatography (100% MeCN).
